# EspC, an Autotransporter Protein Secreted by Enteropathogenic *Escherichia coli*, Causes Apoptosis and Necrosis through Caspase and Calpain Activation, Including Direct Procaspase-3 Cleavage

**DOI:** 10.1128/mBio.00479-16

**Published:** 2016-06-21

**Authors:** Antonio Serapio-Palacios, Fernando Navarro-Garcia

**Affiliations:** Department of Cell Biology, Centro de Investigación y de Estudios Avanzados del IPN (CINVESTAV-IPN), México City, Mexico

## Abstract

Enteropathogenic *Escherichia coli* (EPEC) has the ability to antagonize host apoptosis during infection through promotion and inhibition of effectors injected by the type III secretion system (T3SS), but the total number of these effectors and the overall functional relationships between these effectors during infection are poorly understood. EspC produced by EPEC cleaves fodrin, paxillin, and focal adhesion kinase (FAK), which are also cleaved by caspases and calpains during apoptosis. Here we show the role of EspC in cell death induced by EPEC. EspC is involved in EPEC-mediated cell death and induces both apoptosis and necrosis in epithelial cells. EspC induces apoptosis through the mitochondrial apoptotic pathway by provoking (i) a decrease in the expression levels of antiapoptotic protein Bcl-2, (ii) translocation of the proapoptotic protein Bax from cytosol to mitochondria, (iii) cytochrome *c* release from mitochondria to the cytoplasm, (iv) loss of mitochondrial membrane potential, (v) caspase-9 activation, (vi) cleavage of procaspase-3 and (vii) an increase in caspase-3 activity, (viii) PARP proteolysis, and (ix) nuclear fragmentation and an increase in the sub-G_1_ population. Interestingly, EspC-induced apoptosis was triggered through a dual mechanism involving both independent and dependent functions of its EspC serine protease motif, the direct cleavage of procaspase-3 being dependent on this motif. This is the first report showing a shortcut for induction of apoptosis by the catalytic activity of an EPEC protein. Furthermore, this atypical intrinsic apoptosis appeared to induce necrosis through the activation of calpain and through the increase of intracellular calcium induced by EspC. Our data indicate that EspC plays a relevant role in cell death induced by EPEC.

## IMPORTANCE

EspC, an autotransporter protein with serine protease activity, has cytotoxic effects on epithelial cells during EPEC infection. EspC causes cytotoxicity by cleaving fodrin, a cytoskeletal actin-associated protein, and focal adhesion proteins (i.e., FAK); interestingly, these proteins are also cleaved during apoptosis and necrosis. Here we show that EspC is able to cause cell death, which is characterized by apoptosis: by dissecting the apoptotic pathway and considering that EspC is translocated by an injectisome, we found that EspC induces the mitochondrial apoptotic pathway. Remarkably, EspC activates this pathway by two distinct mechanisms—either by using or not using its serine protease motif. Thus, we show for the first time that this serine protease motif is able to cleave procaspase-3, thereby reaching the terminal stages of caspase cascade activation leading to apoptosis. Furthermore, this overlapped apoptosis appears to potentiate cell death through necrosis, where EspC induces calpain activation and increases intracellular calcium.

## INTRODUCTION

Enteropathogenic *Escherichia coli* (EPEC) infection is a leading cause of infantile diarrhea in developing countries, which can be severe and lethal (1). EPEC elicits a histopathologic lesion formed at the mucosal intestinal surface that displays a pedestal-like structure, known as an attaching and effacing (AE) lesion (2). The genes responsible for the AE phenotype are located in a 35.6-kb pathogenicity island termed the locus of enterocyte effacement (LEE) (3), and the LEE is organized into five polycistronic operons (LEE1 to -5). LEE1, LEE2, and LEE3 encode a type III secretion system (T3SS), or injectisome. LEE4 comprises the T3SS-secreted proteins EspA, EspB, and EspD (EPEC-**s**ecreted **p**rotein), which are also components of this translocation apparatus by which other effector proteins are translocated into the cell. Thus, a LEE5 effector, Tir, is injected by the T3SS directly into the cell and is inserted in the membrane, exposing an extracellular domain that is recognized by intimin (an EPEC membrane adhesin). Intimin-Tir interaction leads to intimate adherence and pedestal formation beneath adherent bacteria (4). Other LEE effector proteins are also injected into the cell (EspG, EspZ, EspH, Map, and EspF) during infection (5, 6). Notably, there are also non-LEE-encoded effectors in EPEC that are translocated by the T3SS, including NleA/EspI, EspJ, EspL, EspO NleB, NleC, NleD, NleE, NleF, NleG, NleH, and Cif (cycle inhibiting factor) (7). All of these effectors (LEE and non-LEE) interfere with different aspects of the cell physiology, including subverting innate immune pathways, specifically those involved in phagocytosis, host cell survival, apoptotic cell death, and inflammatory signaling, which are all required to cause disease (8, 9).

EPEC encodes a subset of effectors that promote cell death, including EspF, Map, and Cif. EspF is associated with several phenotypes, including increased intrinsic apoptotic cell death and caspase-dependent loss of epithelial growth factor receptor (6, 10, 11). EspF is imported into the mitochondrial matrix by the host machinery due to a canonical N-terminal mitochondrial targeting sequence (10). Thus, EspF destabilizes mitochondrial membrane potential (ΔΨ_m_), leading to cytochrome *c* release, caspase activation, and downstream intrinsic apoptosis (10, 12, 13). Nougayrède et al. have shown that EspF also interacts with Abcf2, which appears to inhibit caspase activation in intestinal epithelial cells, facilitating Abcf2 degradation and potentially leading to increased activation of caspases-3 and -9 and promoting intrinsic apoptotic cell death (14). Map effector also uses an N-terminal mitochondrial targeting sequence (13, 15, 16) to subsequently induce disruption of the mitochondrial membrane potential and cytochrome *c* release and trigger intrinsic apoptosis (15–18). However, Map is also associated with morphological changes in mitochondria, suggesting differences between Map and EspF activities (16, 18). The cycle inhibiting factor (Cif) effector has no known impact on mitochondrial function but was found to arrest cell cycle progression at G_2_/M, G_1_/S, or both G_2_ and G_1_ transitions (19–23). However, unlike the other two effectors, prolonged infection is needed for the cell cycle arrest. Moreover, Cif induces delayed apoptosis, as indicated by the accumulation of activated caspase-3 and increased caspase activation at 24, 48, and 72 h postinfection (24). Both phenotypes require Cif deamidase activity (23–25). Interestingly, EPEC also injects T3SS effectors inhibiting cell death. Apoptosis is a form of programmed cell death that can proceed through two pathways: an extrinsic, receptor-mediated pathway and an intrinsic, mitochondrion-mediated pathway (26). Thus, several EPEC T3SS effectors interfere with host apoptotic pathways, such as NleD and NleB1/NleB2 for the extrinsic pathway and NleH1/NleH2, EspZ, EspT, and EspM (the latter two by inhibiting EspH) for the intrinsic pathway, as well as NleF, which directly inhibits caspases involved in both apoptotic pathways (8, 27). All of these data suggest EPEC’s ability to antagonize host apoptosis during infection through promotion and inhibition of effectors injected by the T3SS; thus, many other effectors could play a role in driving to a final outcome. However, the overall functional relationships between these effectors during infection are poorly understood.

A second pathogenicity island of EPEC that encodes EspC has been identified in pathogenic EPEC1 strains. Unlike proteins secreted by the T3SS, EspC secretion is mediated by the type V secretion system (T5SS), or autotransporter system (28, 29). A recent study showed that *espC* is one of the most prevalent genes among those encoding autotransporter proteins in both typical and atypical EPEC strains (30). Interestingly, EspC is not efficiently internalized under nonphysiological conditions (as a purified protein), because no receptor is involved in its uptake. However, EspC physiologically secreted by EPEC is efficiently internalized during the interaction of EPEC and epithelial cells (31). Recently, we also showed that during EPEC infection, EspC is secreted from the bacteria by T5SS and can then be efficiently translocated into epithelial cells by the T3SS translocon (32).

This efficient EspC delivery into the cell leads to a cytopathic effect characterized by cell rounding and cell detachment that depends on the EspC internalization and its functional serine protease motif, as detected by using purified EspC protein (33) or EspC-secreting EPEC (34). The cell detachment phenotype is triggered by cytoskeletal and focal adhesion disruption. EspC produced by EPEC is able to cleave fodrin, paxillin, and focal adhesion kinase (FAK). *In vitro* assays using purified proteins showed that EspC directly cleaves these cytoskeletal and focal adhesion proteins, while a point mutation engineered in its serine protease catalytic site abolishes this activity. The kinetics of protein degradation indicated that EspC first cleaves fodrin (within the 11th and 9th repetitive units at the Q1219 and D938 residues), and this event sequentially triggers paxillin degradation, FAK dephosphorylation, and FAK degradation. Thus, cytoskeletal and focal adhesion protein cleavage lead to the cell rounding and cell detachment promoted by EspC-producing EPEC (34). Interestingly, endogenous proteins such as caspases and calpains also cleave fodrin and FAK during cell death (35), and the morphological features of these dying cells are similar to those seen in EspC-treated cells, including cell shrinkage and blebs (33). Here, we show that EspC is able to cause programed cell death through the intrinsic or mitochondrial apoptosis pathway and hijacks this pathway by using its serine protease motif to directly cleave procaspase-3. Interestingly, EspC is also able to cause necrosis by inducing activation of calpain by increasing intracellular calcium.

## RESULTS

### EspC is involved in the cell death caused by EPEC.

**FIG 1:**
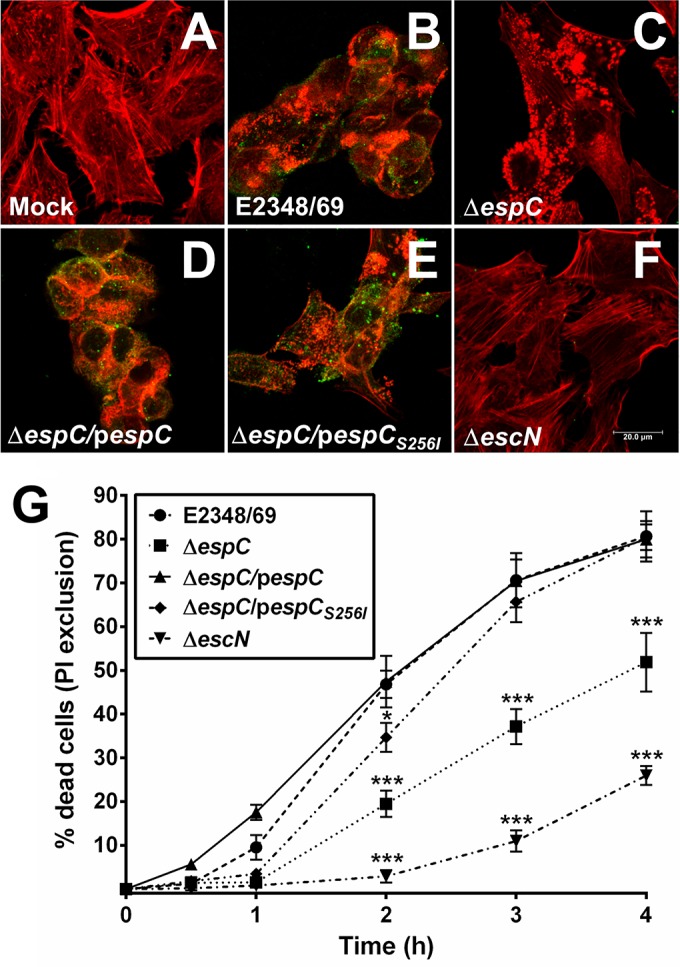
EspC produces cell death on epithelial cells. (A to F) Cytotoxic effects induced by EspC-producing EPEC. Untreated HEp-2 cells were used as the control (A), and HEp-2 cells were infected with the EPEC wild-type (WT) strain (E2348/69) (B), Δ*espC* mutant (C), Δ*espC*/p*espC* complemented strain (D), Δ*espC*/p*espC_S256I_* complemented strain (E), or Δ*escN* mutant (F) for 4 h. Infected cells were fixed and permeabilized. Cells were immunostained with anti-EspC antibody, followed by a secondary antibody conjugated to fluorescein isothiocyanate (FITC), and the actin cytoskeleton was detected with rhodamine-phalloidin. Slides were observed using a Leica TCS SP8 confocal microscope. Scale bar, 20 µm. (G) Cell death induced by EspC-producing EPEC. HEp-2 cells were infected with the EPEC WT, Δ*espC*, Δ*espC*/p*espC*, Δ*espC*/p*espC_S256I_*, or Δ*escN* strain at an MOI of 10 for different lengths of time. Cells were harvested and stained with propidium iodide (PI) to perform a PI exclusion assay by flow cytometry. Data are expressed as the mean ± SEM from at least 3 independent experiments. Statistical analysis was performed using two-way ANOVA followed by Dunnett’s multiple comparison test for comparison to the WT strain (*, *P* < 0.05; ***, *P* < 0.001).

We have previously shown that EspC is able to cause cytotoxic effects due to the cleavage of fodrin and then focal adhesion proteins (34). Cell rounding and detachment represent accurate parameters to describe these cytotoxic effects. However, it is unknown if these rounded cells are in the process of cell death and if the detached cells are dead. To further understand the relationship between cytotoxicity and cell death caused by EspC, we sought the correlation between the cytotoxic effects and cell death caused by EspC by using EPEC wild-type (WT) strain E2348/69, an *espC* mutant (Δ*espC*) strain, and the complemented (Δ*espC*/p*espC*) strain. A T3SS mutant (Δ*escN*) strain was used as the negative control as it is unable to translocate EspC inside the cells. HEp-2 cells infected with the Δ*escN* strain for 4 h showed no cytotoxic effects (Fig. 1F) and appeared similar to uninfected cells (Fig. 1A). Cells infected with the EPEC wild type (E2348/69) showed the classical cytotoxic effects (Fig. 1B), characterized by cytoskeleton contraction, cell rounding, and cell detachment as previously reported (33). Interestingly, cells infected with the Δ*espC* mutant showed no cytotoxic effects, and some cells displayed normal actin stress fibers but with formation of many pedestals along the cells (Fig. 1C), which is followed by cell rounding, but with much reduced cell detachment in comparison to cells infected with the wild type strain (which contains the *espC* gene). In contrast, the complemented (Δ*espC*/p*espC*) strain was able to cause cytotoxic effects similar to the wild type (Fig. 1D). Interestingly, the complementation of the mutant with a site-directed mutation in the serine protease motif of EspC (Δ*espC*/p*espC_S256I_*) caused similar damage to the Δ*espC* strain (Fig. 1E), suggesting an important role played by the enzymatic activity in the development of cell toxicity. (In this complemented strain, p*espC_S256I_* is a plasmid carrying the *espC* gene that encodes a change from serine to isoleucine at amino acid position 256.)

We investigated the cell death in these rounded cells under the same conditions but now also quantifying cell death in detached cells (all of the cell culture). To do this, the remaining attached cells were recovered using Accutase solution, and all detached and recovered cells were analyzed by flow cytometry using propidium iodide (PI) at 0.5, 1, 2, 3, or 4 h of infection with the different bacterial strains used above. Infection with either the wild-type strain or the complemented *espC* mutant (Δ*espC*/p*espC*) strain caused higher cell death than infection with the Δ*espC*/p*espC_S256I_*, Δ*espC*, or Δ*escN* strain in that order of magnitude. The Δ*espC*/p*espC* strain initially caused more cell death than the wild-type strain at 1 and 2 h, but after 2 h, both strains followed the same cell death kinetics until both reached 80% dead cells. The Δ*espC* strain did not cause cell death up until 1 h of infection, but a time-dependent increase was observed starting at 2 h and reaching 50% dead cells after 4 h. The Δ*espC*/p*espC_S256I_* strain caused a cell death percentage intermediate between those of the wild-type and Δ*espC* strains but reached a similar cell death percentage (80%) to the wild type at 4 h of infection (Fig. 1G). On the other hand, in cells treated with the Δ*escN* mutant, cell death was only detected at 3 and 4 h of interaction, to reach around 25% dead cells (Fig. 1G). All of these conditions were also tested in HT-29 cells with similar results (see Fig. S1A in the supplemental material). Thus, the cytotoxic effects caused by EspC were related to cell death (Fig. 1G).

### EspC induces apoptosis and necrosis in epithelial cells.

**FIG 2:**
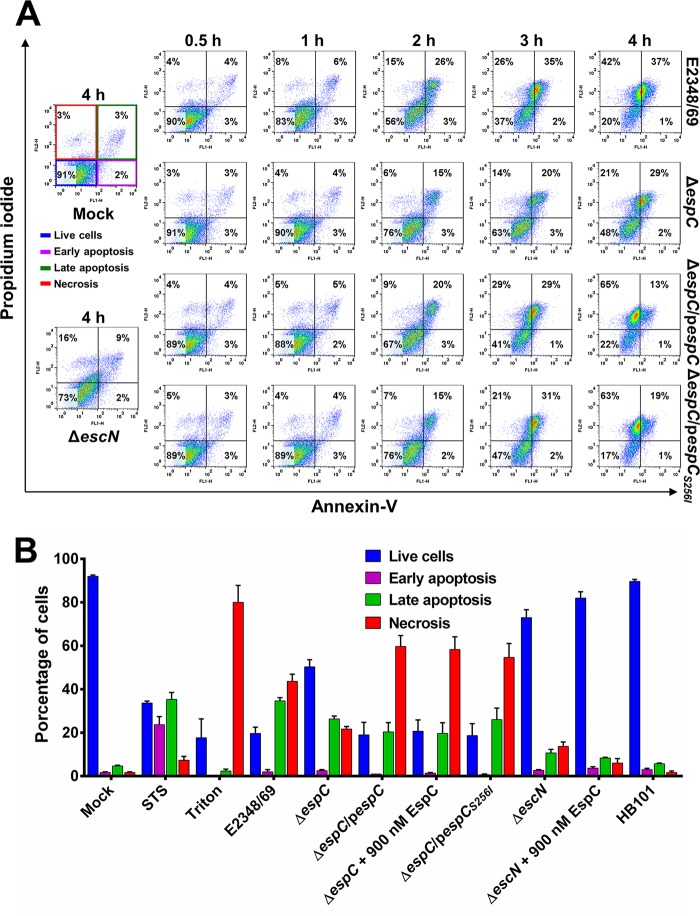
EspC induces apoptosis and necrosis on epithelial cells. (A) Analyses of apoptotic and necrotic cells during kinetics of infection. FACS analysis via annexin V and PI staining was used to observe the induction of apoptosis and necrosis of HEp-2 cells infected by the EPEC WT, Δ*espC*, Δ*espC*/p*espC*, Δ*espC*/p*espC_S256I_*, or Δ*escN* strain at an MOI of 10 for the indicated lengths of time. Mock-infected cells were used as a negative control. Annexin V-negative and PI-negative cells represent live cells. Annexin V-positive and PI-negative cells represent the early apoptotic populations. Annexin V-positive and PI-positive cells represent the late apoptotic populations. Annexin V-negative and PI-positive cells represent the necrotic populations. Representative flow cytometric dot plots are shown. (B) Distribution of the infected cell populations at 4 h of infection. HEp-2 cells were infected with the bacterial strains for 4 h as indicated. Mock-infected cells were used as a negative control. Staurosporine (STS) at 1 µM and 0.1% Triton X-100 were used as positive controls for apoptosis or necrosis, respectively. The results were plotted and are shown as the mean ± SEM from at least 3 independent experiments.

Given that propidium iodide quantification allowed for the determination of the total percentage of cell death regardless of whether the cells died via an apoptotic or necrotic pathway, we decided to investigate how EspC induces cell death by determining what types of cell death mechanisms are involved and which is the first one to be triggered. Initially we sought to define if EspC was involved in cell death by apoptosis or necrosis at different time points from 30 min to 4 h. Epithelial cells (HEp-2 and HT-29) were infected with different strains and then stained with propidium iodide and annexin V to be analyzed by flow cytometry. Based on this type of analysis, it is possible to detect stained cells associated with four different phenotypes described by four quadrants: normal cells, cells in early apoptosis, cells in late apoptosis, and necrotic cells (Fig. 2A). Cells infected with the Δ*escN* strain, which is unable to translocate T3SS effectors, including EspC, displayed (at 4 h of interaction) 73% live cells versus 91% in untreated cells (mock cells), 2% early apoptotic cells similar to mock cells, 9% late apoptotic cells versus 3% in mock cells, and 16% necrotic cells versus 3% in mock-infected cells. The EPEC wild-type strain provoked cell death, causing both apoptosis and necrosis (Fig. 2A; see Fig. S1B in the supplemental material). The proportion of apoptotic cells increased with the EPEC wild-type infection time: mainly the late apoptotic cells went from 4% at 30 min to 37% at 4 h, while early apoptosis was kept at 1% to 3% of cells in all times analyzed and all treatments used. On the other hand, the proportion of necrotic cells also increased with infection time by the wild-type strain: from 4% at 30 min to 42% at 4 h. Thus, at 30 min the EPEC-infected cells were similar to 4-h mock-infected cells with regard to apoptotic and necrotic cells. The initial fastest increase for late apoptotic cells started at 2 h of infection, reaching 26%, while the increase for necrotic cells started at 3 h of infection, also reaching 26% (Fig. 2A), showing that apoptosis proceeds to necrosis. Interestingly, the proportion of both apoptotic and necrotic cells decreased in cells infected with the Δ*espC* strain in comparison with those cells infected with the wild type. The proportion of apoptotic cells decreased according to infection time with respect to the proportion reached in wild-type-infected cells; the percentages of late apoptotic cells were 26 and 37% at 2 and 4 h, respectively, in wild-type-infected cells, while the percentages in Δ*espC* strain-infected cells were 15 and 29% at the same infection times. Similarly, the percentages of necrotic cells induced by the Δ*espC* strain were smaller than those of cells infected with the wild-type strain at both 2 and 4 h of infection (WT infection, 15 and 42%, versus Δ*espC* infection, 6% and 21%, respectively). As expected, the Δ*espC* mutant strain complemented with the *espC* gene recovered the cell death phenotype, but the kinetics was different from that seen in cells infected with the wild-type strain. For instance, necrotic cells increased quickly from 9% at 2 h to 29% at 3 h to reach the highest dead cell population of 65% at 4 h, while the apoptotic cells increased from 20% at 2 h to only 29% at 3 h, and then this population decreased to 13% at 4 h of infection. Interestingly, the strain complemented with *espC_S256I_* reached similar percentage values of necrotic cells to the strain complemented with the native *espC* gene but only at 4 h of infection, while the kinetics at early times were different and the percentages were slightly lower (i.e., Δ*espC*/p*espC* strain infection, 9 and 29% at 2 and 3 h versus Δ*espC*/p*espC_S256I_* strain infection, 7 and 21% at the same infection times). All of these data indicate that (i) EspC is involved in the cell death caused by the EPEC WT (E2348/69) and (ii) EspC is able to induce apoptosis and necrosis in epithelial cells. Additionally, these data suggest that apoptosis could be the first event that can lead to increased necrosis.

To further understand the cell death induced by EspC, apoptotic and necrotic cells were analyzed with infection with a wide variety of strains and various conditions at 4 h of infection (Fig. 2B). To further standardize the detection system of apoptotic and necrotic cells using annexin V and propidium iodide, two additional controls were selected: staurosporine (STS) and Triton X-100. As expected, STS increased the early and late apoptosis populations (24 and 35% compared to 2 and 5% in mock-infected cells), whereas Triton X-100 increased the necrotic cell population (80% compared to 2% in mock-infected cells). Both reagents caused a decrease in live cells (33 and 18%, respectively, compared to 91% in mock-infected cells) (Fig. 2B). Under this condition, EPEC caused increases in late apoptotic and necrotic cells of 35 and 43%, whose population rates decreased to 25 and 21% when the Δ*espC* mutant was used. As expected, complementation of the Δ*espC* mutant with the gene or 900 nM exogenous EspC (which uses the T3SS to translocate EspC into the cell) restored and increased cell death by necrosis up to 59 to 58%, at the expense of a decrease of the late apoptotic cells to 20 and 19%, respectively. In contrast, the addition of 900 nM exogenous EspC did not complement the Δ*escN* mutant, and the results were similar to those detected in mock-infected cells and in the cells infected with the Δ*escN* mutant alone. Interestingly, the complementation induced by the *espC* gene in the Δ*espC* background had similar effects to complementation done by using the *espC_S256I_* gene, suggesting that the catalytic activity of EspC may not be involved in the cell death.

### EspC induces caspase-3 activity.

**FIG 3:**
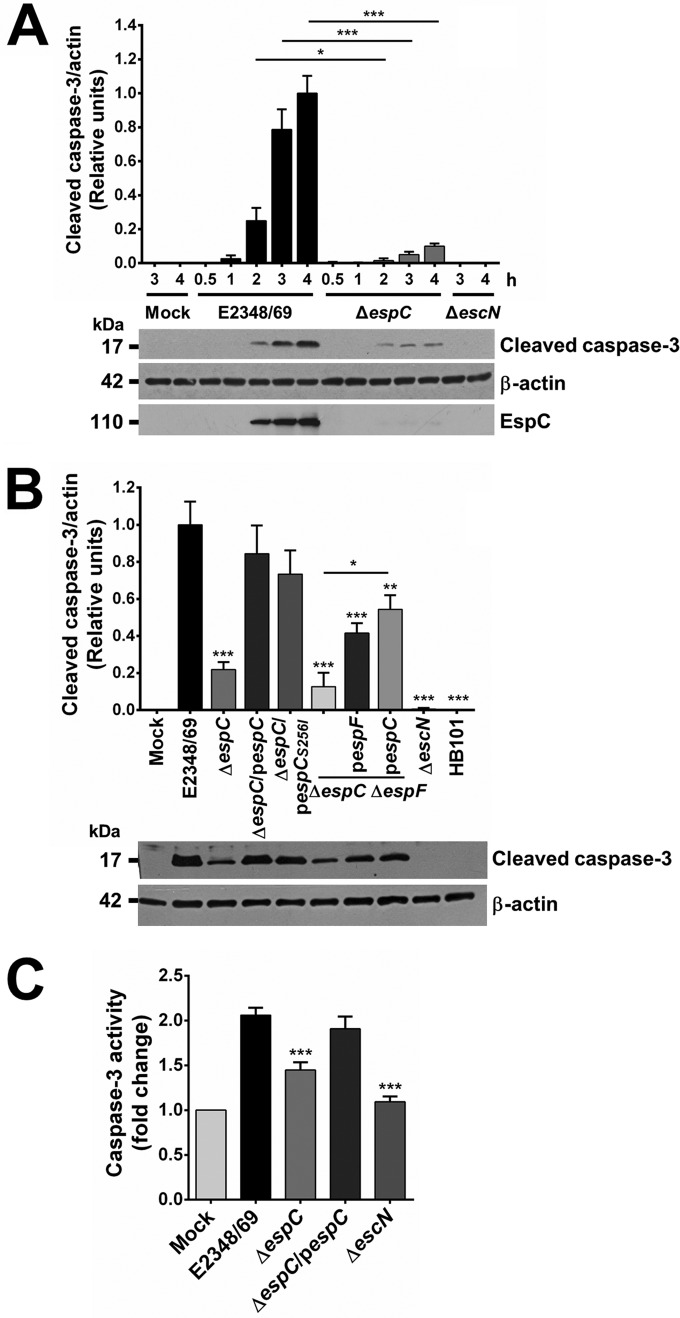
EspC is required for caspase-3 activation and activity. (A) EspC-producing EPEC induces procaspase-3 cleavage. HEp-2 cells were infected with the indicated bacterial strains at an MOI of 10 at the different times indicated. Infected cells were lysed, and proteins were analyzed by immunoblotting using anti-caspase-3, anti-β-actin, and anti-EspC as primary antibodies and HRP-conjugated anti-isotype secondary antibody. Samples were normalized using the maximum densitometric value. (B) Induction of procaspase-3 cleavage is complemented in the mutant strains by either *espC* or *espF*, and this effect is higher by *espC* complementation. Activation of caspase-3 was performed as indicated above, in cells infected by the indicated strains at 4 h of infection. (C) EspC-producing EPEC induces caspase-3 activity. Activity of caspase-3 was determined in HEp-2 cells infected by the EPEC WT, Δ*espC*, Δ*espC*/p*espC*, or Δ*escN* strain at an MOI of 10 for 4 h. The whole-cell lysates were subjected to the caspase activity assay by using a synthetic substrate as described in Materials and Methods. Activity is represented as fold change relative to uninfected cells. Data are shown as the mean ± SEM from at least 3 independent experiments. Statistical analyses were performed using (A) unpaired *t* test (*, *P* < 0.05; ***, *P* < 0.001) or (B and C) one-way ANOVA followed by Dunnett’s multiple comparison test for comparison to the WT strain (*, *P* < 0.05; **, *P* < 0.01; ***, *P* < 0.001).

Since cell death induced by EspC appeared to be initiated first through apoptosis, we decided to investigate if EspC was involved in the activation and activity of caspase-3, the main executioner caspase involved in apoptosis process. Epithelial cells were infected with the EPEC wild-type strain or the Δ*espC* mutant. Uninfected cells (mock-infected cells) or cells infected with a T3SS mutant (Δ*escN*) strain were used as negative controls. Caspase-3 activation was detected as cleaved caspase-3, a 17-kDa subproduct of procaspase-3, by Western blotting using anti-caspase-3 antibodies. Bands of activated caspase-3 were normalized using actin as a housekeeping protein, and the presence of EspC in the infected cells was detected using anti-EspC antibodies. EPEC was able to induce the cleavage of procapase-3 from 2 h of infection, and the cleaved caspase-3 band increased as a function of infection time, while the Δ*espC* mutant was unable to induce efficient cleavage of procaspase-3 given that the detection of the cleaved caspase-3 band was very faint. Mock-infected cells and cells infected with the Δ*escN* strain did not allow detection of the cleaved caspase-3 band (Fig. 3A).

The unequivocal detection of caspase-3 activation at 4 h prompted us to investigate the role of EspC in this process within this time window by using the different bacterial strains indicated above to infect both the HEp-2 and HT-29 cell lines. The Δ*espC* mutant clearly displayed only 20% of caspase-3 activation compared with the EPEC wild type. As expected, complementation of the Δ*espC* mutant with the *espC* gene recovered caspase-3 activation to 84%, and activation was not statistically different from that of the wild type. Interestingly, the Δ*espC* mutant complemented with *espC* gene mutated in the serine protease motif (Δ*espC*/p*espC*_S256I_) caused the same magnitude of caspase-3 activation as the strain complemented with the native *espC* gene (Fig. 3B; see Fig. S1C in the supplemental material).

It is well known that the multifunctional T3SS effector EspF from EPEC, but not an *espF* mutant, is able to induce caspase-3 cleavage (10, 14). To investigate the role of EspC versus the role of EspF in the activation of caspase-3, we constructed a double mutant (Δ*espC* Δ*espF*) strain, and its effects on procaspase-3 were compared to those of strains complemented with either *espC* or *espF*. Both genes were able to increase levels of cleaved casapase-3 in comparison with the double mutant, but neither of them reached the wild-type or Δ*espC*/p*espC* levels (Fig. 3B). Cleaved caspase-3 was undetectable in the Δ*escN* mutant, similarly to untreated conditions or cells infected with nonpathogenic strain *E. coli* HB101, which suggests that other T3SS effectors could be acting in this pathway.

In order to verify if the processing of procaspase-3 to cleaved caspase-3 contributes to the activity of caspase-3, we quantified the activity of caspase-3 on Ac-DEVD-AMC (acetyl Asp-Glu-Val-Asp-7-amido-4-methylcoumarin), a specific substrate for this caspase, in cells infected by EPEC WT, Δ*espC*, or Δ*espC*/p*espC* strains for 4 h. Mock-infected cells and cells infected with the Δ*escN* strain were used as negative controls. The EPEC WT caused a 2-fold increase in caspase-3 activity compared to mock-infected cells, while the Δ*espC* mutant did not reach such activity levels but was significantly different from the wild type. This decrease in activity level observed in cells infected with the Δ*espC* mutant was recovered when the cells were treated with the *espC*-complemented strain to values similar to those reached by the wild-type strain (Fig. 3C). As expected, the Δ*escN* mutant did not induce caspase-3 activity, and its activity values were similar to those of the mock-infected cells.

### EspC stimulates the intrinsic mitochondrial apoptosis pathway.

**FIG 4:**
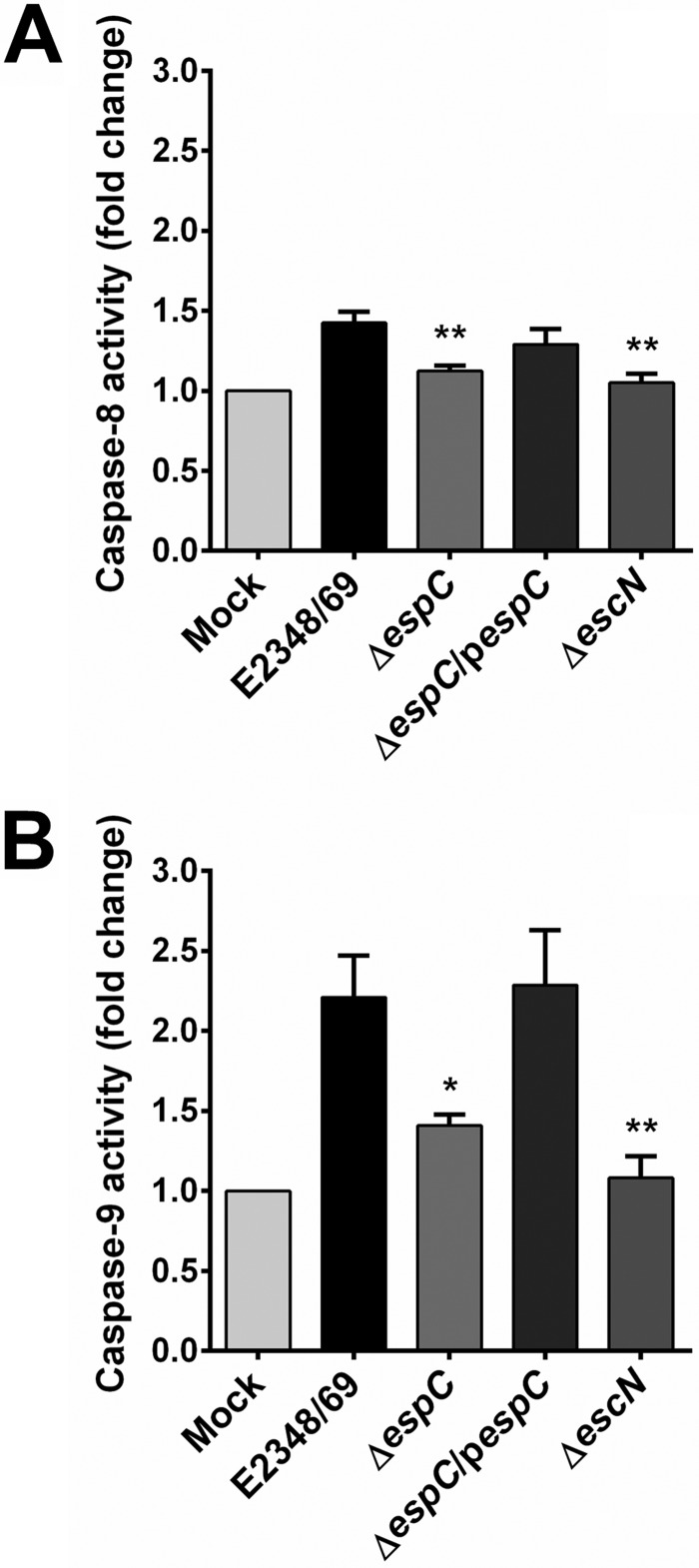
EspC preferentially induces caspase-9 activity. (A) Activity of caspase-8 and (B) caspase-9 induced by EspC. HEp-2 cells were infected with the EPEC WT, Δ*espC*, Δ*espC*/p*espC*, or Δ*escN* strain at an MOI of 10 for 4 h. The whole-cell lysates were subjected to caspase activity assay by using specific substrates for each caspase as described in Materials and Methods. Activity is represented as fold change relative to uninfected cells. Data are expressed as the mean ± SEM from at least 3 independent experiments. Statistical analysis was performed using one-way ANOVA followed by Dunnett’s multiple comparison test for comparison to the WT strain (*, *P* < 0.05; **, *P* < 0.01).

In order to determine which apoptotic pathway is stimulated by EspC during the infection of epithelial cells by EPEC, we quantified the activities of caspase-8 and caspase-9 by using their specific substrates, Ac-IETD-AFC (acetyl-Ile-Glu-Thr-Asp-7-amino-4-triflouromethylcoumarin) and Ac-LEDH-AFC (acetyl-Leu-Glu-His-Asp-7-amino-4-trifluoromethylcoumarin), respectively. These caspases are differentially activated during the extrinsic or intrinsic pathway, respectively. The EPEC wild-type and *espC*-complemented strains were able to induce a modest caspase-8 activity, while the Δ*espC* mutant was unable to induce this activation, thus reaching similar levels to those in mock-infected cells or cells infected with the Δ*escN* mutant (Fig. 4A). Remarkably, the wild-type and the *espC*-complemented strains induced a 2-fold increase in caspase-9 activity, while the Δ*espC* and Δ*escN* strains were unable to cause a similar significant increase (Fig. 4B).

**FIG 5:**
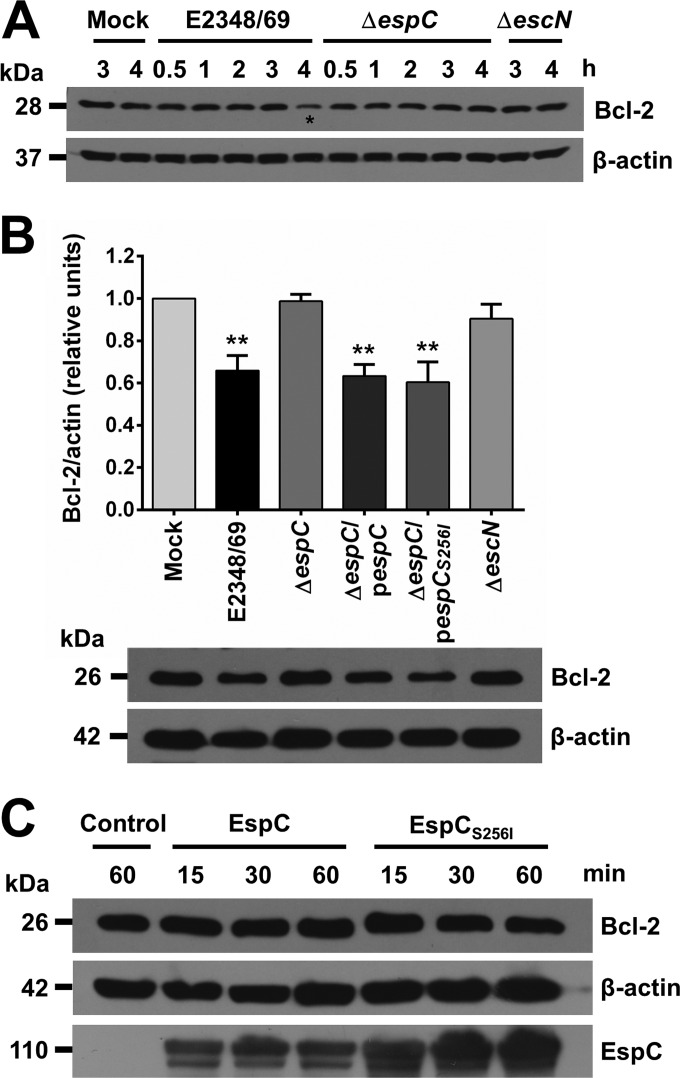
EspC induces a decrease in Bcl-2 protein levels. (A) Kinetics of detection of Bcl-2 in cells infected with the EPEC WT and Δ*espC* mutant. HEp-2 cells were infected with bacterial strains as indicated and for the indicated lengths of time. The asterisk indicates a clear Bcl-2 decrease induced by EPEC (B) detection of Bcl-2 at 4 h of infection with the EPEC WT, Δ*espC*, Δ*espC*/p*espC*, Δ*espC*/p*espC_S256I_*, or Δ*escN* strain at an MOI of 10. Infected cells were lysed, and proteins were analyzed by immunoblotting using anti-Bcl-2 and anti-β-actin as primary antibodies and HRP-conjugated anti-isotype secondary antibody. Densitometries of three immunoblots were plotted. Results are expressed as the mean ± SEM from at least 3 independent experiments. Statistical analysis was performed using one-way ANOVA followed by Dunnett’s multiple comparison test for comparison with mock-infected cells (**, *P* < 0.01). (C) EspC is unable to cleave Bcl-2. Uninfected HEp-2 cells were lysed, and protein extracts were incubated with purified EspC or EspC_S256I_ for 15, 30, and 60 min. Reactions were stopped with Laemmli buffer, and products were separated by SDS-PAGE, transferred to a PVDF membrane, and analyzed by immunoblotting using anti-Bcl-2, anti-β-actin, and anti-EspC as primary antibodies and HRP-conjugated anti-isotype secondary antibody.

Caspase-3 and caspase-9 activation induced by EspC suggested a process of caspase-3 activation through formation of the cytochrome *c*/Apaf-1/caspase-9-containing apoptosome complex (36). Caspase activation is closely linked to permeabilization of the outer mitochondrial membrane by proapoptotic members of the Bcl-2 family (37). In order to investigate if EspC-induced apoptosis causes a decrease in antiapoptotic Bcl-2 protein expression, cells infected with either the EPEC wild-type or Δ*espC* strain for different lengths of time (30 min to 4 h) were analyzed by Western blotting using anti-Bcl-2 antibodies. The wild-type strain but not the Δ*espC* mutant caused a decrease in Bcl-2 protein band intensity at 4 h of infection, while no effect was detected for the Δ*espC* mutant nor for the Δ*escN* mutant, with levels comparable to those of mock-infected cells (Fig. 5A). The wild-type infection-generated decrease in Bcl-2 protein band intensity was recorded as 40% in comparison to infection with the Δ*espC* mutant or the negative control and cells infected with the Δ*escN* mutant (Fig. 5B). Infection with the Δ*espC* mutant complemented with either *espC* or *espC_S256I_* also decreased the Bcl-2 protein band intensity at wild-type levels, suggesting that the serine protease motif could not be require for this mechanism. To further explore the role of the serine protease motif of EspC in the decrease in Bcl-2 protein band intensity, which might be caused through processing by EspC, purified EspC or EspC_S256I_ was added to uninfected lysed cells and the proteolysis was analyzed by Western blotting using anti-Bcl-2 antibodies. However, neither the addition of EspC nor that of EspC_S256I_ was able to induce Bcl-2 cleavage (Fig. 5C).

**FIG 6:**
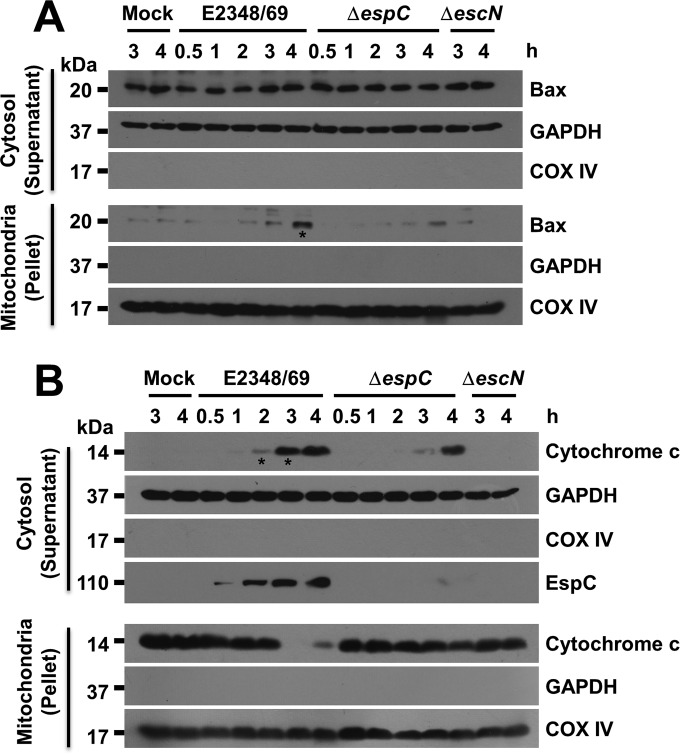
EspC stimulates Bax translocation to mitochondria and cytochrome *c* release. EspC induces Bax translocation at 4 h of infection (A) and release of cytochrome *c* from mitochondria to cytosol (B). HEp-2 cells were infected with the different strains as indicated at an MOI of 10 and for the indicated lengths of time. Host cell cytosolic and mitochondrial fractions were separated and analyzed by immunoblotting using (A) anti-Bax or (B) anti-cytochrome *c* as primary antibodies and HRP-conjugated anti-isotype secondary antibody. Blots were also probed for COX IV as a control marker for the mitochondrial fractions and GAPDH as a control marker for the cytosolic fractions. The blots shown are representative of three independent experiments. Asterisks indicate a clear increase induced by EPEC.

Altogether, these data suggested that Bax, the proapoptotic protein, must be translocated to mitochondria at 4 h of infection by EPEC, which would lead to cytochrome *c* release from the mitochondria, an event that would be absent in the case of the Δ*espC* mutant. To test this hypothesis, epithelial cells infected for different time periods with the wild-type strain or Δ*espC* mutant were fractionated to obtain cytosolic and mitochondrial fractions, and Bax translocation was detected by Western blotting using anti-Bax antibodies. Anti-GAPDH (anti-glyceraldehyde-3-phosphate dehydrogenase) and anti-COX IV antibodies were used as fractionation markers for cytosol and mitochondria, respectively. The translocation of Bax into the mitochondria was detected at 4 h during EPEC infection, but not during Δ*espC* mutant infection (Fig. 6A), perfectly correlating with the Bcl-2 data. Furthermore, by using the same approach, we identified cytochrome *c* release from mitochondria and its detection in the cytosol fraction by Western blotting using anti-cytochrome *c* antibodies. The EPEC wild type caused a clear cytochrome *c* release from the mitochondria starting at 2 h of infection, and at 3 and 4 h, mitochondria were practically depleted from cytochrome *c* (Fig. 6B). These results strongly correlated with time-dependent cytochrome *c* detection in the cytosolic fraction starting at 2 h of infection (Fig. 6B). Interestingly, cytochrome *c* was clearly detected starting at 4 h in cells infected with the Δ*espC* mutant, but this detection did not correlate with the depletion of cytochrome *c* from mitochondria, suggesting again the participation of other effectors since this effect was completely lacking in cells infected with the Δ*escN* mutant, which showed results similar to those of the mock-infected cells (Fig. 6B).

**FIG 7:**
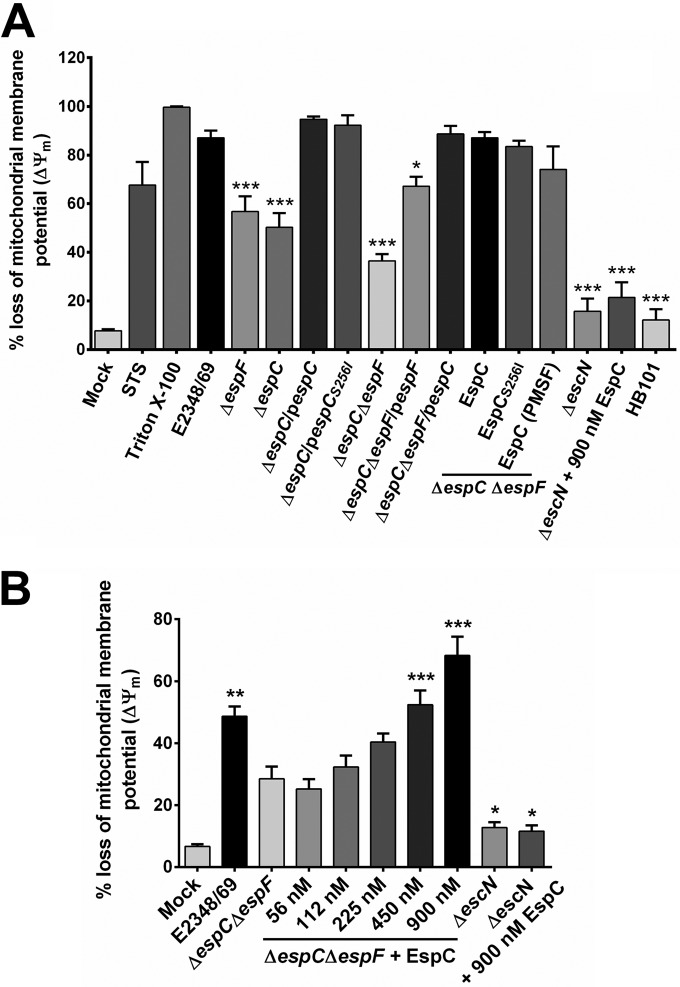
EspC induces depolarization of mitochondrial inner membrane (ΔΨ_m_). (A) EspC is involved in the loss of mitochondrial membrane potential. HEp-2 cells were prestained with rhodamine 123 and then infected with bacterial strains as indicated at an MOI of 10 for 4 h. Purified EspC, EspC_S256I_, or EspC (900 nM) preincubated with PMSF was added during the infection to complement the double mutant as indicated. Staurosporine (STS) and Triton X-100 were used as positive controls. All cells were analyzed for the loss of mitochondrial inner membrane potential (ΔΨ_m_) by flow cytometry. (B) Complementation of the EPEC Δ*espC* Δ*espF* strain with exogenous EspC. HEp-2 cells were infected with bacterial strains as indicated and supplemented with increasing molarities of purified EspC at an MOI of 10 for 3 h. Cells were processed and analyzed as indicated above. Data are expressed as the mean ± SEM from at least 3 independent experiments. Statistical analysis was performed using one-way ANOVA followed by Dunnett’s multiple comparison test for comparison to the (A) WT or (B) Δ*espC* Δ*espF* strain (*, *P* < 0.05; **, *P* < 0.01; ***, *P* < 0.001).

These data suggested that EspC must induce a loss of mitochondrial membrane potential (ΔΨ_m_), a role reminiscent of the one reported for the effector EspF (10). Thereby, we investigated the role of EspC and its serine protease motif in the induction of loss of ΔΨ_m_ in cells under different treatments (Fig. 7A; see Fig. S1D in the supplemental material) and compared these effects to those caused by EspF. The mitochondrial membrane potential was recorded by prestaining epithelial cells with rhodamine 123, a cell-permeable dye that is sequestered by active mitochondria that have a negative membrane potential. Treatment with 2% Triton X-100 was set at 100% of ΔΨ_m_ loss, given its dramatic effect. EPEC infection caused an 87% loss in ΔΨ_m_ at 4 h, while Δ*espC* strain infection produced a 50% loss in ΔΨ_m_ and cells with mock infection displayed an 8% loss. As expected, the *espC*-complemented strain recovered the phenotype, showing a 95% loss in ΔΨ_m_, and a similar recovery was displayed when the complementation *espC* gene comprised a point mutation in its serine protease motif. In fact, these percentages were slightly higher than wild-type levels (Fig. 7A; see Fig. S1E). Interestingly, an *espF* mutant caused a 56% loss in ΔΨ_m_, slightly higher than the Δ*espC* mutant, whereas the double mutant (Δ*espC* Δ*espF*) strain caused a 36% loss in ΔΨ_m_. Moreover, the double mutant complemented with *espF* displayed 67% loss in ΔΨ_m_, whereas *espC* complementation supplanted the former, with levels similar to those of the wild type at 89%. To confirm the results related to the role of the serine protease motif, epithelial cells were infected with the double mutant that had been complemented with exogenous proteins from EspC, EspC_S256I_, or EspC with the serine protease inhibitor phenylmethylsulfonyl fluoride (EspC-PMSF) for 4 h, which caused 87, 83, and 79% losses in ΔΨ_m_, respectively; values similar to those caused by the Δ*espC* Δ*espF*/p*espC* strain. As expected, the addition of exogenous EspC to the Δ*escN* mutant had no effect on the ΔΨ_m_, and the results were similar to those obtained with the Δ*escN* strain alone or *E. coli* HB101. A careful titration of the exogenous EspC addition assay mixture identified the optimal concentration of 450 nM for efficient concentration-dependent complementation of the double mutant strain (Fig. 7B).

### EspC leads to PARP proteolysis and nuclear fragmentation.

**FIG 8:**
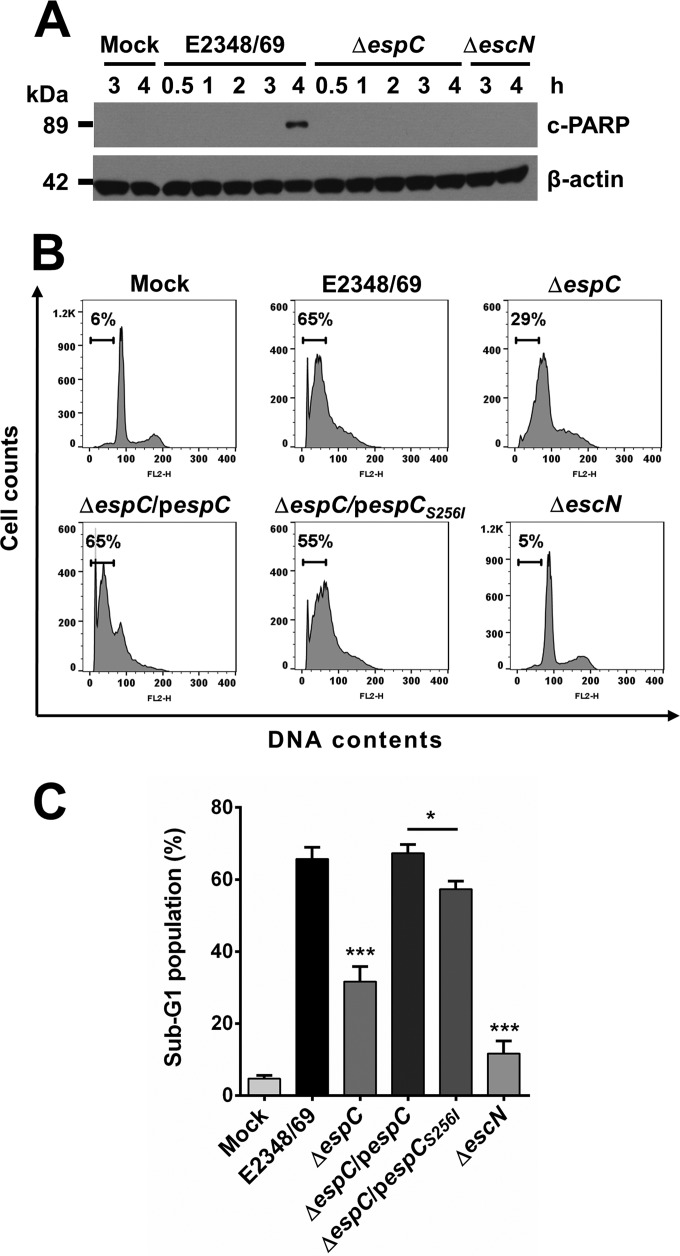
EspC induces PARP proteolysis and DNA nuclear fragmentation. (A) EspC induces PARP cleavage. HEp-2 cells were infected with the bacterial strains as indicated at an MOI of 10 and for the indicated times. Infected cells were lysed, and proteins were analyzed by immunoblotting using anti-cleaved PARP and anti-β-actin as primary antibodies and HRP-conjugated anti-isotype secondary antibody. (B) EspC induces DNA nuclear fragmentation. HEp-2 cells were infected with the EPEC WT, Δ*espC*, Δ*espC*/p*espC*, Δ*espC*/p*espC_S256I_*, or Δ*escN* strain at an MOI of 10 for 4 h. The cell cycle was measured by using propidium iodide (PI) staining; results are presented as histograms. (C) EspC induces an increase in the sub-G_1_ cell population. Cell distribution was measured by fluorescence intensity in the sub-G_1_ phase. Results are shown as percentages of the sub-G_1_ population. Data are expressed as the mean ± SEM from at least 3 independent experiments. Statistical analyses were performed using unpaired *t* test (*, *P* < 0.05) and one-way ANOVA followed by Dunnett’s multiple comparison test for comparison to the WT strain (***, *P* < 0.001).

Poly(ADP-ribose) polymerase-1 (PARP-1), inactivated by caspase-3 cleavage during programmed cell death, plays the active role of “nick sensor” during DNA repair and apoptosis. PARP-1 proteolysis by caspases is concomitant with poly(ADP-ribose) synthesis, and the resulting p89 proteolytic fragment migrates from the nucleus into the cytoplasm in late apoptotic cells with advanced nuclear fragmentation (38). Thereby, we investigated if EspC is able to induce PARP degradation during the infection by EPEC. Epithelial cells were infected with the wild-type strain and Δ*espC* mutant for different lengths of time, and the cleavage of PARP was detected by Western blotting using anti-PARP antibodies. EPEC wild-type infection, but not Δ*espC* mutant infection, was able to produce the expected 89-kDa proteolytic fragment from cleaved PARP (c-PARP) at 4 h. Like infection with the Δ*espC* mutant, infection with the negative controls, mock infection, and infection with the Δ*escN* mutant were unable to induce cleavage of PARP at any of the times tested (Fig. 8A).

These last results suggested that EspC might contribute to DNA fragmentation occurring in the apoptosis. To test this hypothesis, epithelial cells were infected with the wild type, Δ*espC* mutant, and strains complemented with the native *espC* gene or the *espC* catalytic site point mutant for 4 h, and cell fragmented DNA was analyzed by flow cytometry using propidium iodide. In mock-infected cells, the fragmented DNA represented 6% of the cell population, and this level was similar for cells treated with the Δ*escN* mutant, used as a negative control (Fig. 8B). EPEC caused an increase in the population with fragmented DNA, which reached 65%, while the Δ*espC* mutant caused a more modest increase, reaching 29%. As expected, the *espC*-complemented strain recovered the ability to cause DNA fragmentation at similar level (65%) to the wild type. Interestingly, the *espC_S256I_*-complemented strain recovered this phenotype at a level slightly lower (55%) than the wild type (Fig. 8B). In fact, the analysis of three independent experiments revealed that the wild-type strain increased the sub-G_1_ population to 66%, versus the 31% induced by the Δ*espC* mutant. The strain complemented with the native *espC* gene yielded a complete recovery up to wild-type levels but not the *espC* gene mutated in the serine protease active site (Fig. 8C).

### EspC can induce apoptosis independent of or dependent on initiator caspases.

**FIG 9:**
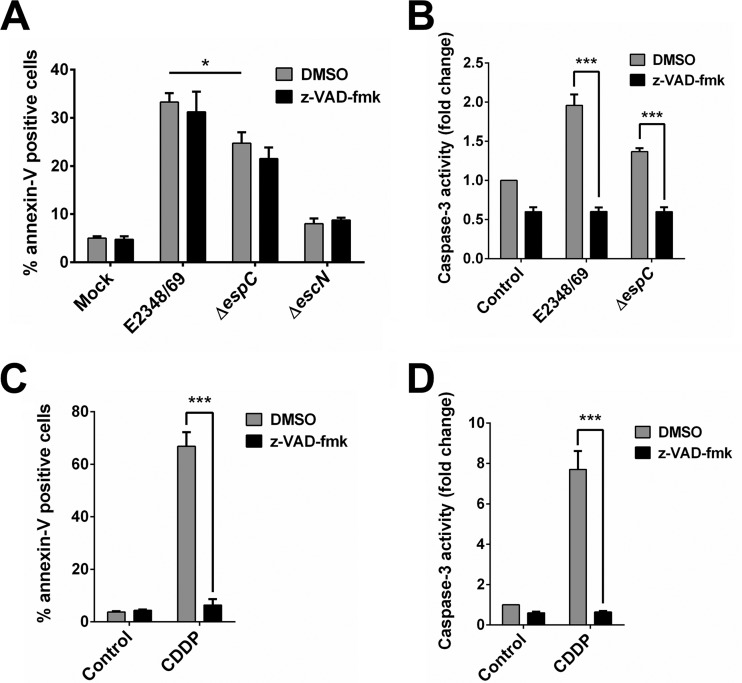
EspC also induces caspase-independent cell death. (A and C) Caspase inhibition does not block apoptosis induced by EPEC. HEp-2 cells were pretreated with 50 µM z-VAD-fmk or only DMSO for 1 h. Cells were infected with the EPEC WT, Δ*espC*, or Δ*escN* strain at an MOI of 10 for 4 h (A) or treated with 100 µM cisplatin (CDDP) for 18 h (C). Mock-infected cells were used as a negative control. Cells were harvested and labeled with annexin V and analyzed by flow cytometry. Annexin V-positive cells were considered apoptotic cells. (B and D) Caspase inhibition blocks caspase-3 activity induced by EPEC. Activity of caspase-3 was determined in HEp-2 cells pretreated with 50 µM z-VAD-fmk or only DMSO for 1 h and infected with the EPEC WT or Δ*espC* strain at an MOI of 10 for 4 h (B) or 100 µM CDDP for 18 h (D). The whole-cell lysates were subjected to the caspase-3 activity assay as described in Materials and Methods. Activity is represented as fold change relative to uninfected cells. Data are expressed as the mean ± SEM from at least 3 independent experiments. Statistical analysis was performed using two-way ANOVA followed by Bonferroni’s multiple comparison test (*, *P* < 0.05; ***, *P* < 0.001).

To better understand the role of the serine protease motif of EspC on the apoptosis induced by EPEC, we blocked initiator caspases to monitor apoptosis and caspase-3 activity. First of all, epithelial cells were preincubated with the caspase inhibitor z-VAD-fmk [*N*-benzyloxycarbonyl-Val-Ala-Asp(O-Me) fluoromethyl ketone] and then infected with the wild-type, Δ*espC*, and Δ*escN* strains and compared with untreated cells. Cells were simultaneously analyzed for apoptosis using annexin V and for caspase-3 activity using the Ac-DEVD-AMC substrate. A canonical caspase inducer, cisplatin (CDDP), was used in conjunction with z-VAD-fmk to validate the inhibitor’s functional effect on caspase-3 activity and annexin V. Surprisingly, the apoptosis induced by the wild-type strain and by the Δ*espC* mutant was not inhibited by the caspase inhibitor (Fig. 9A). Interestingly, the caspase inhibitor did cause an inhibition of the caspase-3 activity induced by either the wild-type or Δ*espC* strain to reach mock-infected cell levels (Fig. 9B). As expected, the caspase inhibitor prevented both the apoptosis and the caspase-3 activity induced by CDDP (Fig. 9C and D). These and previous data indicate that EspC can cause apoptosis by a mechanism both dependent on and independent of initiator caspases and suggest that other proteases could be cleaving caspase-3, possibly through the serine protease activity of EspC.

### EspC is able to directly cleave caspase-3.

**FIG 10:**
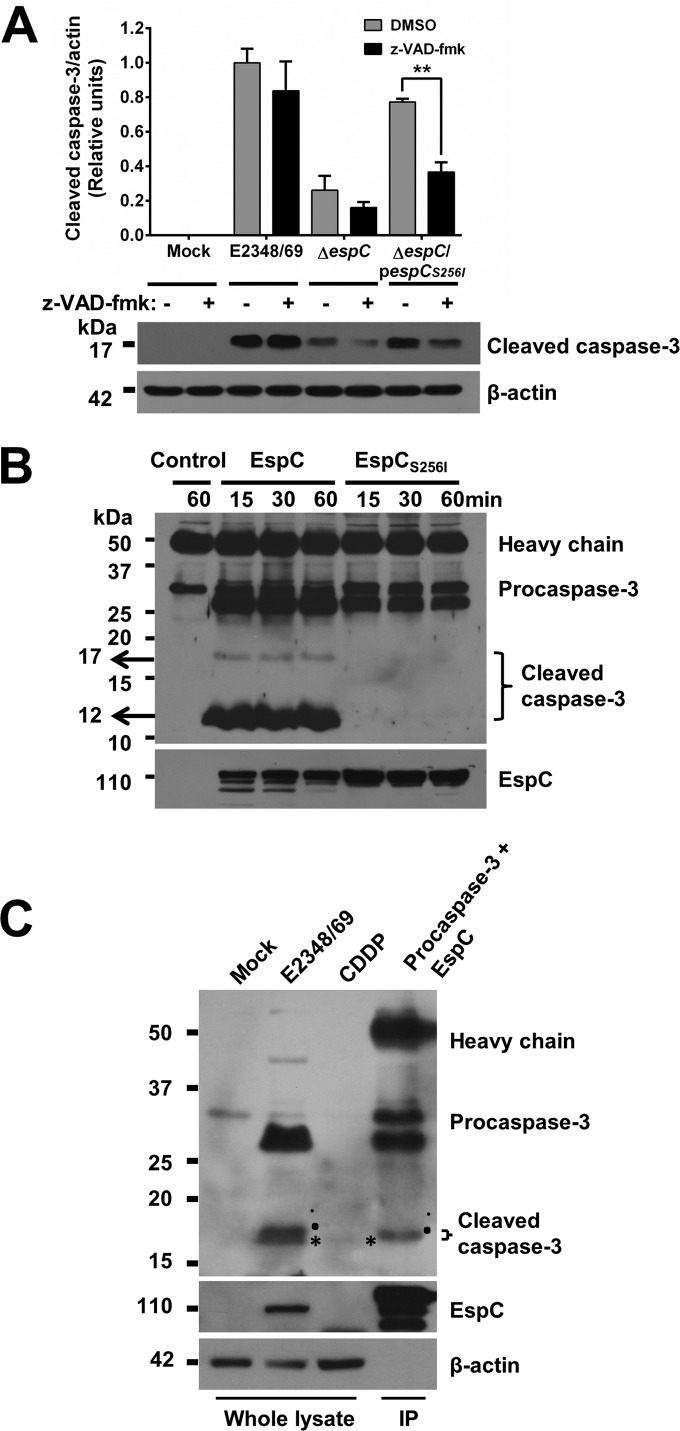
Procaspase-3 is directly cleaved by EspC. (A) The presence of a caspase inhibitor plus a mutation in the serine protease motif causes a dramatic decrease in caspase-3 activation. HEp-2 cells were pretreated with 50 µM z-VAD-fmk or only DMSO for 1 h. Cells were infected with the EPEC WT, Δ*espC*, or Δ*espC*/p*espC_S256I_* strain at an MOI of 10 for 4 h. Infected cells were lysed, and proteins were analyzed by immunoblotting using anti-caspase-3 and anti-β-actin as primary antibodies and HRP-conjugated anti-isotype secondary antibody. Data are expressed as the mean ± SEM from at least 3 independent experiments. Statistical analysis was performed using two-way ANOVA followed by Bonferroni’s multiple comparison test (**, *P* < 0.01). (B) Purified EspC cleaves procaspase-3. Uninfected HEp-2 cells were lysed, and protein extracts were incubated with the primary antibody anti-caspase-3 plus protein A-agarose overnight. Immunoprecipitated procaspase-3 was incubated with purified EspC or EspC_S256I_ for 15, 30, and 60 min. Reactions were stopped with Laemmli buffer, and products were separated by SDS-PAGE, transferred to a PVDF membrane, and analyzed by immunoblotting using anti-caspase-3 as a primary antibody and HRP-conjugated anti-isotype secondary antibody. Heavy chains of antibodies used for the immunoprecipitation were used as a loading control. (C) Differential cleavage of procaspase-3 by EspC and caspases. HEp-2 cells were infected with the EPEC WT at an MOI of 10 for 4 h or treated with 100 µM cisplatin (CDDP) for 18 h and were processed as indicated above. Additionally immunoprecipitated procaspase-3 was incubated with purified EspC as indicated above. All samples were analyzed by immunoblotting using anti-caspase-3, anti-EspC, and anti-β-actin as primary antibodies and HRP-conjugated anti-isotype secondary antibody. Asterisks indicate fragments of about 17 kDa, and dots indicate fragments of about 18 kDa.

To determine if the serine protease activity of EspC is involved in the cleavage of caspase-3, epithelial cells were preincubated or not with z-VAD-fmk and then infected with the wild-type strain, Δ*espC* mutant, or the mutant complemented with *espC* mutated in the serine protease motif. The cleavage activity on procaspase-3 was monitored by detecting the cleaved caspase-3 band by Western blotting using anti-caspase-3 antibodies. As expected and according to the data on inhibition of the caspase-3 activity, the caspase inhibitor was unable to inhibit the cleavage of procaspase-3 in cells treated with the wild-type strain since similar levels of cleaved caspase-3 were detected in cells pretreated or not with the inhibitor. Remarkably, the cleavage activity on procaspase-3 that was lost in the Δ*espC* mutant was recovered by the complementation with *espC* mutated in the serine protease motif and was inhibited by the caspase inhibitor, in contrast to wild-type strain infection (Fig. 10A).

To further confirm the procaspase-3 cleavage results and to know if EspC is able to directly cleave procaspase-3 by using its serine protease motif, procaspase-3 was immunoprecipitated from epithelial cells using anti-caspase-3 antibodies and incubated with recombinant EspC or EspC_S256I_ proteins for different lengths of time (15, 30, and 60 min). The cleavage of procaspase-3 to produce cleaved caspase-3 was detected by Western blotting using anti-caspase-3 antibodies. Excitingly, native EspC was able to cleave procaspase-3 to produce a subproduct of about 17 kDa (the enzymatically active cleaved caspase-3) and another of 12 kDa (confirming the heterodimer), while the site-directed *espC* mutant was unable to cleave procaspase-3, since only the intact protein band was detected (Fig. 10B).

In light of this finding, we carefully reanalyzed our previous detection of cleaved caspase-3 by Western blotting (i.e., see Fig. 3), and we noticed a doublet band of cleaved caspase-3 in cells treated with the wild-type strain, thus suggesting that procaspase-3 might be cleaved at different sites by either EspC or initiator caspases (in this case, caspase-9). It is noteworthy that the higher-molecular-mass band disappeared in cells infected with the Δ*espC* mutant but was detected in cells infected with the *espC*-complemented strain. To better differentiate these two bands, we further increased their differential electrophoretic mobility by switching from 12% to 15% SDS-PAGE and compared the cleavage of procaspase-3 in epithelial cells infected with the wild-type strain versus immunoprecipitated procaspase-3 treated with the purified EspC protein; as a positive control, cells treated with CDDP were used. By using this approach, it was possible to define two separated bands from the cleaved caspase doublet band in EPEC-treated cells and only one (the bigger one) processed by EspC, while the lower band was similar to that produced by CDDP, an inductor of initiator caspases (Fig. 10C). These data indicate that EspC is able to directly cleave procaspase-3 in a site different from but very close to the cleavage site of the initiator caspases.

### Role of EspC in cell death by necrosis.

**FIG 11:**
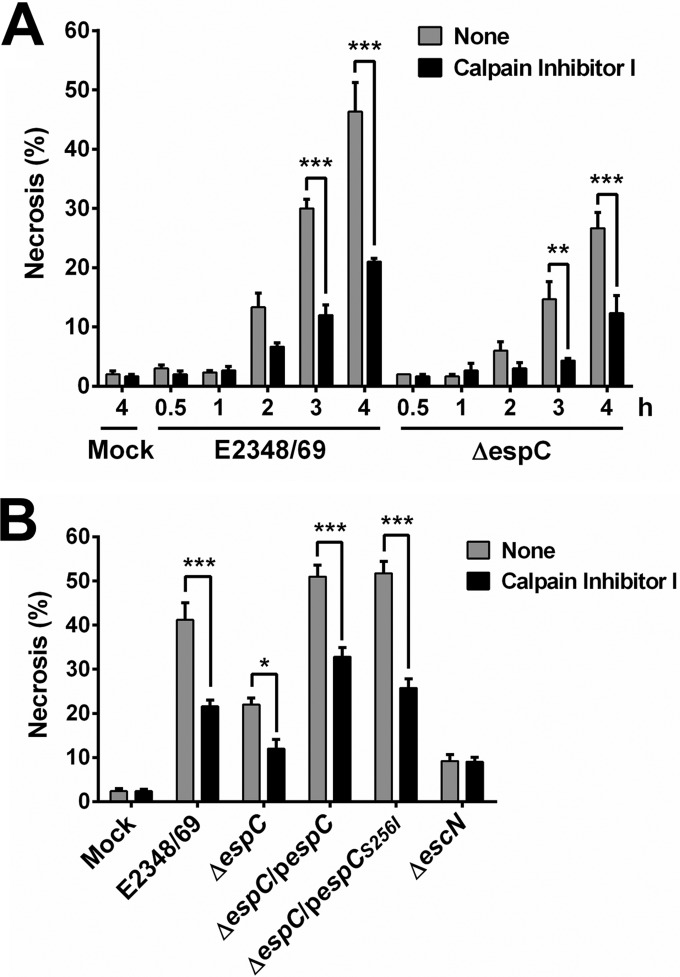
EspC induces necrosis through calpain activation. (A) Kinetics of necrosis induction by the EPEC WT and Δ*espC* mutant and their inhibition by a calpain inhibitor. HEp-2 cells were pretreated with 50 µM calpain inhibitor I for 1 h or untreated. Cells were infected with either the EPEC WT or Δ*espC* mutant at an MOI of 10 for the indicated times. Cells were harvested, labeled with annexin V/PI, and analyzed by flow cytometry. Annexin V-negative and PI-positive cells were considered necrotic cells. (B) Comparison of cells infected with the different bacterial strains for 4 h. Cells were infected with the indicated strains, processed, and analyzed as indicated above. Data are shown as the mean ± SEM from at least 3 independent experiments. Statistical analyses were performed using (A) unpaired *t* test (**, *P* < 0.01; ***, *P* < 0.001) or (B) two-way ANOVA followed by Bonferroni’s multiple comparison test (*, *P* < 0.05; ***, *P* < 0.001).

As shown in Fig. 2, EspC is also involved in the cell death by necrosis. To further understand this process, epithelial cells were infected with the wild-type strain (E2348/69), Δ*espC* mutant, and the complemented strains with the native *espC* gene or the site-directed *espC* mutant (Δ*espC*/p*espC* or Δ*espC*/p*espCS_256I_*) for different lengths of time (from 30 min to 4 h); the T3SS mutant (Δ*escN*) was used as a control. Infected cells were analyzed in the cell necrosis quadrant for cells stained with annexin V and propidium iodide and compared with cells that were previously treated with the calpain inhibitor I, an inhibitor of calpain-induced necrosis. The wild-type strain was able to induce necrosis starting at 2 h of infection (13.3% versus 2% in mock cells), and this effect increased with time (30% at 3 h and 46.3% at 4 h), while the Δ*espC* mutant only induced necrosis starting at 3 h of infection (14.6%), and this effect increased to 26.6% at 4 h. In both infections (wild type or Δ*espC* mutant), calpain inhibitor I was able to inhibit at almost 50% at each time tested (Fig. 11A). Thus, by testing the different strains at 4 h of infection, it was clear that either the lack of EspC or the treatment with calpain inhibitor I caused a 50% decrease in the necrosis induced by the wild-type strain (from about 40% to about 20%). Moreover, the increase of 20% induced by Δ*espC* strain infection was further decreased to 10% by calpain inhibitor I. Interestingly, the complemented strains with *espC* or *espC_S256I_* caused increases of necrosis of 51 and 51.7%, respectively, which were higher than that of the wild-type strain (41.2%), and these values decreased to 32.8 and 25.7%, respectively, when calpain inhibitor I was used (Fig. 11B); similar data were obtained by using MDL28170 (data not shown).

### EspC induces calpain activity that is dependent on intracellular calcium.

**FIG 12:**
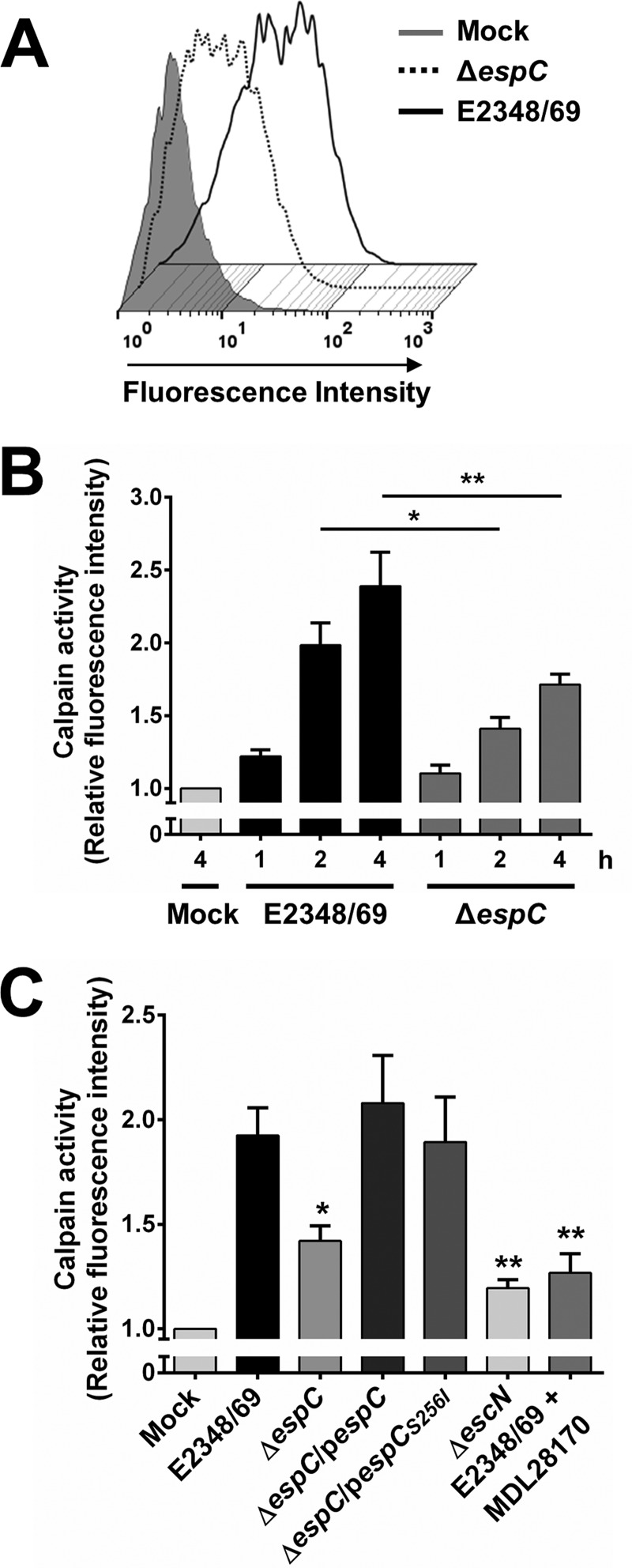
EspC induces calpain activity during EPEC infection. (A) Measurement of calpain activity induced by EspC. HEp-2 cells were pretreated with 20 µM fluorescent substrate *t*-BOC-Leu-Met-CMAC upon cleavage by calpain. Cells were infected with the EPEC WT (E2348/69) or Δ*espC* mutant at an MOI of 10 for 4 h. Calpain activity was measured as increase in fluorescence intensity. (B) Kinetics of calpain activity induced by the EPEC WT or Δ*espC* mutant. Cells were infected with the bacterial strains at an MOI of 10 for the indicated times. Calpain activity was measured as increase of mean fluorescence intensity. All measurements were expressed relative to the calpain activity measured in mock-infected cells. Activity is represented as fold change relative to uninfected cells. (C) EspC causes an increase in calpain activity. HEp-2 cells were infected with the different bacterial strains as indicated at an MOI of 10 for 3 h. MDL28170, a specific calpain inhibitor, was included to demonstrate that the increase of fluorescence is specific to calpain-like proteases. Data are shown as the mean ± SEM from at least 3 independent experiments. Statistical analyses were performed using (B) unpaired *t* test (*, *P* < 0.05; **, *P* < 0.01) or (C) one-way ANOVA followed by Dunnett’s multiple comparison test for comparison to the WT strain (*, *P* < 0.05; **, *P* < 0.01).

The previous data suggest that EspC is able to induce calpain activity. To test this, epithelial cells were first loaded with the synthetic substrate for calpain *t*-BOC-Leu-Met-CMAC (*t*-BOC-Leu-Met-7-amino-4-chloromethylcoumarin) and were then infected with the different strains. The fluorescence intensity was detected by flow cytometry. By using this approach, it is possible to detect calpain substrate cleavage by monitoring the fluorescence intensity inside the cells, which is denoted by a logarithmic displacement along the fluorescence intensity axis (Fig. 12A). Thus, in the kinetics of infection (1, 2, and 4 h) with the wild type, the fluorescence intensity increased with the infection time and these values were significantly lower in cells infected with the Δ*espC* mutant (Fig. 12B). A systematic comparison of the different strains at 3 h of infection (to avoid the leaking of calpain substrate induced by the complemented strains at 4 h of infection due to the high percentage of necrotic cells) revealed that the calpain activity induced by Δ*espC* strain infection was 50% lower than that by infection with the wild type and that complementation with either *espC* or *espC_S256I_* could rescue the calpain activity to wild-type levels (Fig. 12C). As an internal control, we used MDL28170, a specific inhibitor of calpain, which was able to inhibit the calpain activity of the wild-type strain to around 71% (almost to the levels induced by the Δ*escN* strain).

**FIG 13:**
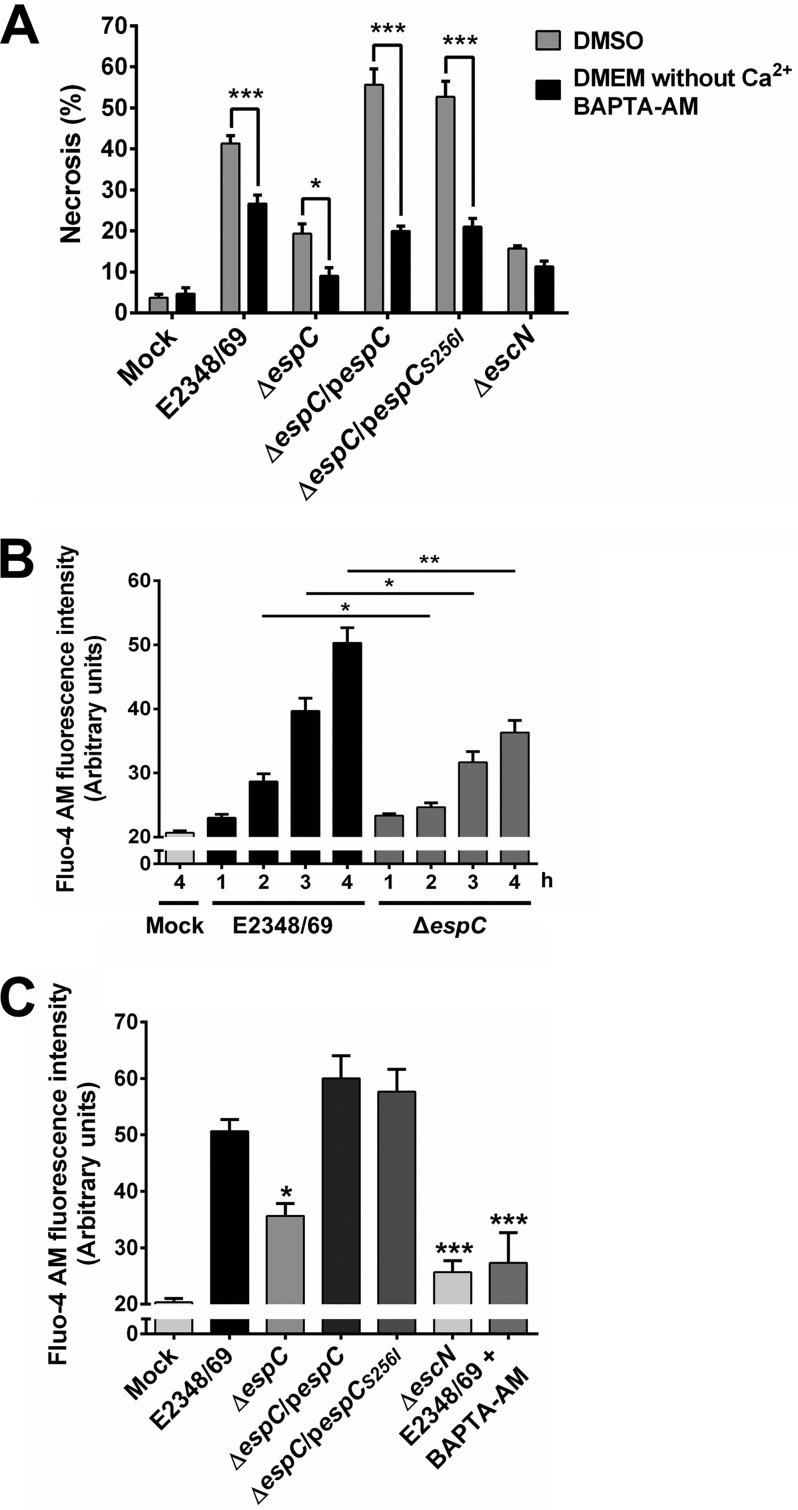
EspC induces an increase in intracellular Ca^2+^ associated with necrosis. (A) Intracellular calcium chelation causes a decrease in the necrosis induced by EspC. HEp-2 cells were pretreated with 20 µM BAPTA-AM or only DMSO for 1 h. HEp-2 cells were infected with the indicated bacterial strains at an MOI of 10 for 4 h. Cells were harvested and labeled with annexin V and PI and analyzed by flow cytometry. Annexin V-negative and PI-positive cells were considered necrotic cells. Data are shown as the mean ± SEM from at least 3 independent experiments. Statistical analysis was performed using two-way ANOVA followed by Bonferroni’s multiple comparison test (*, *P* < 0.05; ***, *P* < 0.001). (B) Kinetics of increase of intracellular calcium induced by the EPEC WT and Δ*espC* strains. HEp-2 cells were pretreated with 4 µM Fluo-4 AM for 1 h. Cells were infected with either the EPEC WT or Δ*espC* mutant for the indicated times. Cells were harvested, and the mean Fluo-4 AM fluorescence intensity of each condition was analyzed by flow cytometry. (C) The presence of EspC increases intracellular calcium. HEp-2 cells were treated with the indicated bacterial strains for 4 h. BAPTA-AM, an intracellular calcium chelator, was used as a negative control. Cells were pretreated and treated as indicated above. Data are shown as the mean ± SEM from at least 3 independent experiments. Statistical analyses were performed using (B) unpaired *t* test (*, *P* < 0.05; **, *P* < 0.01) or (C) one-way ANOVA followed by Dunnett’s multiple comparison test for comparison to the WT strain (*, *P* < 0.05; ***, *P* < 0.001).

In order to investigate the role of the calcium in relation to calpain activity and the necrosis induced by EspC, epithelial cells were pretreated with Dulbecco’s modified Eagle’s medium (DMEM) without calcium but supplemented with the calcium chelator BAPTA-AM, and the infected cells were analyzed by flow cytometry in the quadrant of necrosis. In these cells, intracellular calcium chelation also inhibited the necrosis at around 50% compared with the level in cells infected with the wild-type and Δ*espC* strains without BAPTA pretreatment (Fig. 13A). Interestingly, BAPTA-AM caused a more efficient decrease in the necrosis induced by the complemented strains (with *espC* or *espC_S256I_*), even though they cause the maximum percentage of necrosis.

To confirm these data, we quantified the intracellular calcium in kinetics of infection with the wild-type and Δ*espC* strains. Epithelial cells were previously loaded with Fluo-4 AM, and then the infected cells were analyzed by flow cytometry. Infection with the wild-type strain caused an increase in the intracellular calcium, which was time dependent, and the kinetics were similar to those observed for calpain activity, while the Δ*espC* strain was unable to cause a similar increase in the intracellular calcium, and their kinetics were also similar to those of the calpain activity (Fig. 13B). Thus, at 4 h of infection, the Δ*espC* strain was able to increase the intracellular calcium only 50% of that induced by the wild-type strain and *espC* and *espC_S256I_* were able to complement the mutant to induce again an increase higher than the intracellular calcium level induced by the wild type (Fig. 13C). As expected, BAPTA-AM was also able to inhibit the increase in the intracellular calcium level induced by the wild-type strain.

## DISCUSSION

EspC is an interesting protein secreted by the T5SS to the extracellular medium by EPEC (28); upon eukaryotic cell contact, EspC is the first protein found in tissue culture medium, and it appears at least 1 h before other T3SS proteins (39). Interestingly, we have previously shown that EspC, once in the extracellular medium, can be translocated inside the cells in a T3SS-dependent fashion (32). Once inside the cells, EspC has enterotoxic as well as cytotoxic effects, and these effects depend on the EspC serine protease motif (33). Furthermore, the cytotoxic effects characterized by cell rounding and detachment are due to the cleavage of fodrin and then focal adhesion proteins, such as FAK (34). Endogenous proteins also cleave these two proteins during cell death (40–43), and the morphological features of these dying cells are similar to those seen in EspC-treated cells, including cell shrinkage and blebs (33). Here, we have shown that EspC is also involved in the cell death caused by EPEC, and this cell death is correlated with the cytotoxicity induced by EspC-producing EPEC. Furthermore, EspC is able to induce both apoptosis and necrosis in epithelial cells, and the kinetics of infection using wild-type, *espC* mutant (Δ*espC*), and complemented strains showed that apoptosis was the first event, which then led to increased necrosis.

Interestingly, here we show that EspC-induced apoptosis was triggered through both serine protease motif-dependent and -independent mechanisms, and in both cases, apoptosis was mainly induced by the intrinsic mitochondrial apoptotic pathway (44). Thus, we showed that EspC induced a decrease in the antiapoptotic protein Bcl-2 and translocation of the proapoptotic protein Bax from cytosol to mitochondria. As previously demonstrated, this translocation can initiate apoptosis by forming a pore in the mitochondrial outer membrane (45, 46), and indeed we found that this EspC-induced effect allows cytochrome *c* to escape from mitochondria to the cytoplasm. These two latter events correlated with the loss of the mitochondrial membrane potential induced by EspC-producing EPEC and the activation of the proapoptotic caspase cascade, as previously reported in this pathway (44). Indeed, EspC-producing EPEC strains were able to induce mainly caspase-9 activation, but only slightly induced caspase-8—these caspases are activated in either the intrinsic or extrinsic pathway, respectively (47). As is well known, the released cytochrome *c* binds with apoptotic protease activating factor-1 (Apaf-1) and ATP, which then bind to procaspase-9 to create a protein complex known as an apoptosome (48, 49). This apoptosome subsequently cleaves the procaspase to its active form of caspase-9, which in turn activates the effector caspase-3 (50). Thereby, we were able to find activation of caspase-9, which was accompanied by activation of caspase-3 by the cleavage of procaspase-3 by EspC-producing EPEC, but not for the *espC* mutant; these data also coincided with caspase-3 activity on a specific synthetic substrate. This cascade of events allowed us to detect the inactivation of PARP by caspase-3 (51–53), leading to PARP proteolysis induced by EspC-producing EPEC but not by the *espC* mutant. Classically, the p89 proteolytic fragment migrates from the nucleus into the cytoplasm in late apoptotic cells with advanced nuclear fragmentation (38). Thus, we found the p89 fragment in cells infected by EPEC but not in cells infected with the *espC* mutant, while the nuclear fragmentation that leads to an increased sub-G_1_ population was dramatically decreased in cells infected with the mutant. All of these data clearly show that EspC strongly contributes to the induction of the intrinsic mitochondrial apoptosis pathway. As a matter of controversy, it had been reported that EspC is able to decrease the cytotoxicity induced by EPEC infection through a regulation of T3SS-induced pore formation in the host cell membrane (54). However, this appears not to be a general mechanism, because only 48% of EPEC strains (mainly EPEC1 category) contain the *espC* gene (55) and EspC has been associated with strains of high virulence (56). It will be interesting to test the role of EspC in EspB-EspD pore degradation in a normal epithelial cell infection, avoiding many manipulations (54).

Disconcertingly, the *espC* mutant complemented with *espC* recovered all of the previous phenotype of the wild type at 4 h of infection, but the mutant complemented with *espC* harboring a mutation in its active site (*espC_S256I_*) also did so, suggesting no role for the serine protease motif, which is relevant for the cytotoxicity induced by this protein. However, we could detect slight differences between these two complemented strains in kinetics of infection, mainly at early times: among these differences, remarkably the *espC* mutant complemented with *espC_S256I_* was unable to recover the ability to cause DNA fragmentation, like the same mutant complemented with the native *espC* gene, during the apoptosis caused by EPEC. These data suggested us that two events of apoptosis could be occurring, which may overlap. Interestingly, the use of an inhibitor for caspases, z-VAD-fmk, allowed the finding that the blockage of procaspase-3 cleavage inhibited caspase activity but not the EPEC-induced apoptosis, unlike a classical caspase inducer (cisplatin) that blocks both events (57). These data clearly suggested that another factor could be activating caspase-3 to induce apoptosis. Thus, the use of the *espC* mutant complemented with *espC_S256I_* in combination with the caspase inhibitor allowed us to find that the serine protease motif and caspase inhibition cause a dramatic decrease in activated caspase-3 by preventing the cleavage of procaspase-3, indicating that both EspC with the serine protease motif and EspC without it are able to induce procaspase-3 cleavage to the active 17-kDa caspase-3 and suggested that the EspC serine protease motif could be processing procaspase-3. Indeed, excitingly, by using purified proteins, procaspase-3 is proteolytically processed by EspC *in vitro*, but not at all by the mutant with mutation in the serine protease motif, to produce an approximately 17-kDa subproduct similar to, but not the same as, that produced by caspase-9. This is the first evidence of an EPEC virulence factor, EspC, with the ability to cleave procaspase-3 to produce an ≈17-kDa subproduct similar to that produced during caspase-3 activation by endogenous caspases and also the first reported autotransporter protein with the ability to interfere with the caspase cascade for induction of apoptosis. Interestingly, in aging neutrophils, the cleavage and activation of caspase-3 are independent of the canonical caspase-8- or caspase-9-mediated pathway. Instead, caspase-3 activation is mediated by serine protease proteinase 3 (PR3), which is present in the cytosol of aging neutrophils (58). Thus, all of these findings suggest that the apoptosis induced by EspC could occur by direct processing of caspase-3 or through conventional processing by caspase-9 in the intrinsic mitochondrial apoptosis pathway. Probably, this exacerbated apoptosis could be conducive to increased necrosis since apoptosis is preceded by necrosis and in the *espC*-complemented strain the necrosis at late times of infection (4 h) is higher than that in the wild type, or alternatively this modified apoptosis could lead to another kind of cell death, which by its features could be necroptosis, since there was a high depletion of cytochrome *c*, suggesting a high depletion of ATP from mitochondria as well as late (4 h) activation of PARP, which may deplete the stores of cellular NAD^+^ and induce a progressive ATP depletion and necrotic cell death. Both cases can induce necroptosis, because ATP depletion in mitochondria induces this particular kind of cell death (26).

Interestingly, the EPEC wild type is able to cause necrosis in an infection time-dependent fashion, and this phenotype also depends on EspC, but unlike the EspC-induced apoptosis, which is not inhibited by caspase inhibitors, necrosis is blocked by calpain inhibitors. Furthermore, the kinetics of this necrosis induced by the EPEC wild type and the *espC* mutant overlap the kinetics of calpain activity. Additionally, this necrosis is inhibited by chelation of intracellular calcium, and this inhibition, as expected, correlated with calpain inhibition, since calpains are activated by calcium (59). Interestingly, the EPEC wild type is able to increase intracellular calcium, and this increase is time dependent and also depends on intracellular EspC since exogenous EspC, which is translocated inside the cells by the T3SS, does get inside the cells when a mutant with a mutation in the *escN* gene (a mutation that avoids the expression of the T3SS) is used. Furthermore, complementation in *trans* with *espC* or *espC_S256I_* increased the levels of intracellular calcium and necrosis notably more than the levels induced by the wild-type strain. (As we explained before, this occurs at the expense of a decrease in apoptosis compared to the wild type.)

Thus, these data suggest that EspC protein without or with its functional serine protease motif could induce intrinsic mitochondrial apoptosis, probably by causing endoplasmic reticulum (ER) stress (60), which can induce calcium release leading initially to apoptosis and then to increased necrosis. This ER stress could be triggered by the ability of EspC oligomerization, a process recently shown to occur *in vitro* (61), which could be favored by a hallmark feature shared by several autotransporters, which are characterized by a central β-helical stem (62, 63). At the same time, EspC can cause apoptosis by using its serine protease motif to directly process procaspase-3 and initiates apoptosis by using this intracellular shortcut.

## MATERIALS AND METHODS

### Materials.

Cell culture reagents were obtained from Gibco-Invitrogen (Grand Island, NY). Staurosporine, Accutase, calpain inhibitor I, MDL28170, propidium iodide, and rhodamine 123 were purchased from Sigma-Aldrich (St. Louis, MO). Protease inhibitor cocktail tablets (Complete) were purchased from Roche (Hertforshire, United Kingdom). Ac-IETD-AFC (caspase-8 substrate), Ac-LEDH-AFC (capase-9 substrate), and Ac-DEVD-AMC (capase-3 substrate) were acquired from Enzo Life Sciences (Lausen, Switzerland). Rabbit anti-caspase-3 polyclonal antibody (H-227) was purchased from Santa Cruz Biotechnology (Santa Cruz, CA). The dead cell apoptosis kit, rhodamine-phalloidin, Fluo-4 AM, and *t*-BOC-Leu-Met-CMAC were acquired from Invitrogen (Molecular Probes, Eugene, OR). Mouse anti-Bcl-2, mouse anti-Bax, rabbit anti-COX IV, and rabbit anti-cleaved PARP-specific antibodies were purchased from Cell Signaling Technology (Beverly, MA). z-VAD-fmk (pan-caspase inhibitor) and mouse anti-cytochrome *c* monoclonal antibody (clone 7H8.2C12) were purchased from BD Pharmingen (Chicago, IL). Mouse anti-GAPDH (anti-glyceraldehyde-3-phosphate dehydrogenase) monoclonal antibody was acquired from Abcam (Cambridge, United Kingdom).

### Bacterial strains and purification of recombinant proteins.

The characteristics of the strains used in this study are listed in Table 1. All strains were routinely grown in Luria-Bertani (LB) broth or Dulbecco’s modified Eagle’s medium (DMEM) without antibiotics and serum aerobically at 37°C. EPEC cultures were activated for 2 h as previously described (64). To purify EspC recombinant proteins, strain HB101(pJLM174) or HB101(p*espC_S256I_*) (where p*espC_S256I_* is a plasmid carrying the *espC* gene that encodes a change from serine to isoleucine at amino acid position 256) was grown overnight in LB medium plus arabinose (0.2% wt/vol) and ampicillin (100 µg/ml) at 37°C with shaking (180 rpm). Supernatants were obtained by centrifugation at 7,000 × *g* for 15 min, filter sterilized through 0.22-µm-pore filters (Corning, Cambridge, MA), and concentrated 100-fold in an Amicon Ultrafree centrifugal filter device with a 100-kDa cutoff (Millipore, Bedford, MA). Recombinant EspC and the EspC protein with the substitution S256I (EspC_S256I_) were filter sterilized again, aliquoted, and quantified by the Bradford method (33).

**TABLE 1:** Bacterial strains and plasmids used in this study

Strain	Genotype and/or description	Reference(s)
E2348/69 (WT)	Prototype EPEC isolate (O127:H6), *espC*^+^	28, 68
Δ*espC* strain	E2348/69Δ*espC*, *espC* mutant	29
Δ*espC*(p*espC*) strain	E2348/69Δ*espC*(pJLM174), mutant complemented with *espC*	32
Δ*espC*(p*espC_S256I_*) strain	E2348/69Δ*espC*(p*espC_S256I_*), mutant transformed with *espC* mutated at serine 256 for isoleucine	32
Δ*espF* strain	E2348/69Δ*espF*, *espF* mutant	This work
Δ*espC* Δ*espF* strain	E2348/69Δ*espC* Δ*espF*, *espC espF* double mutant	This work
Δ*espC* Δ*espF*(p*espC*) strain	E2348/69Δ*espC* Δ*espF*(pJLM174), double mutant complemented with *espC*	This work
Δ*espC* Δ*espF*(p*espF*) strain	E2348/69Δ*espC* Δ*espF*(p*espF*-His), double mutant complemented with *espF*	This work
CVD452	E2348/69Δ*escN*, T3SS mutant	69
HB101	Nonpathogenic strain, K-12/B hybrid	70
HB101(pJLM174)	Minimal *espC* clone, cloned in pBAD30	28
HB101(p*espC_S256I_*)	*espC* clone mutated in residue S256, with serine substituted for by isoleucine	32

### *espF* mutant and *espC espF* double mutant construction.

To generate an *espF* deletion mutant of EPEC WT (E2348/69) and Δ*espC* strains, the *espF* gene was replaced by a gene encoding kanamycin resistance by use of the λ Red recombinase system (65). The kanamycin resistance gene was amplified from pKD4 by PCR with primers *espF*-FRT-sense (5′-AAT TAG TCA AGC TGT TTC TAC ACT AGG ACG GCA TAT TAC TAG TGC GGC AAT GTA GGC TGG AGC TGC TTC G) and *espF*-FRT-antisense (5′-CCG GGC GGC TTG GCT TAA GAC CTG AAG TAT CAA GAC TTT TCG ATT TTT CAC ATA TGA ATA TCC TCC TTA G). PCR fragments carrying the kanamycin antibiotic resistance gene flanked by regions homologous to the target locus were electroporated into the EPEC wild-type and Δ*espC* mutant carrying the λ Red recombinase expression plasmid pKD46. pKD46 was cured by growing mutant strains at 43°C. Colonies containing the *espF*::Kan knockout were then obtained as described previously (65).

### Culture cells and bacterial infection.

The human epithelial HEp-2 (ATCC CCL23) or HT-29 (ATCC HTB-38) cell lines were cultured in DMEM supplemented with 10% fetal bovine serum (FBS) (HyClone, Logan, UT), 1% nonessential amino acids, 5 mM l-glutamine, penicillin (100 U/ml), and streptomycin (100 µg/ml). Cells were harvested with 0.25% trypsin and 0.53 mM EDTA (Gibco-BRL, Grand Island, NY) in phosphate-buffered saline (PBS [pH 7.4]), resuspended in the appropriate volume of supplemented DMEM, and incubated at 37°C in a humidified atmosphere of 5% CO_2_.

HEp-2 cells grown in 60-mm petri dishes were infected with activated cultures of EPEC (multiplicity of infection [MOI] of 10) or the indicated isogenic mutants with or without purified EspC for the indicated times. HEp-2 cells infected with any mutant or complemented strains were incubated in the presence of l-arabinose (0.2%) and the appropriate antibiotic. Cells were delicately washed three times with ice-cold PBS (pH 7.4) and scraped into a buffer consisting of 20 mM Tris-HCl (pH 7.5), 150 mM NaCl, 2.5 mM sodium pyrophosphate, 1 mM β-glycerophosphate, 1 mM sodium orthovanadate, 1% sodium deoxycholate, 1% IGEPAL CA630, 1 mM EGTA, 1 mM Na_2_EDTA, and 1× Complete protease inhibitor. Then the cells were incubated on ice for 5 min and sonicated briefly (5 s). Whole extracts were centrifuged for 15 min at 21,255 × *g* in a cold microcentrifuge, and the supernatant was used. Protein concentrations were estimated by the Bradford method. Equivalent amounts of proteins were boiled for 5 min, analyzed by sodium dodecyl sulfate-polyacrylamide gel electrophoresis (SDS-PAGE), and electrotransferred to polyvinylidene difluoride (PVDF) membranes for immunoblot analyses essentially as previously described (34), using anti-caspase-3, anti-cleaved PARP, anti-Bcl-2, anti-EspC, and anti-β-actin antibodies.

### Confocal microscopy.

Eight-well LabTek slides (VWR, Bridgeport, NJ) were seeded with HEp-2 cells at a density of 35,000 cells/well. Before infection with activated EPEC or derivatives, cells were washed three times with DMEM without supplements and incubated at 37°C for 1 h. Infections were carried out for 4 h. Infected HEp-2 cells were washed with PBS, fixed with 4% paraformaldehyde–PBS for 20 min, washed, permeabilized by the addition of 0.1% Triton X-100–PBS for 10 min, and stained with a 1:120 dilution of rhodamine-phalloidin and with a 1:80 dilution of polyclonal rabbit anti-EspC, as previously described (33), followed by an anti-rabbit fluorescein-labeled antibody. Slides were mounted with Vectashield mounting medium (H-1000; Vector Laboratories, Inc.), covered with glass coverslips, and examined under a Leica TCS SP8 confocal microscope.

### Fluorometric analysis of caspase activity.

HEp-2 cells cultured in 35-mm plates were lysed with 400 µl of caspase lysis buffer containing 50 mM HEPES, 5 mM DTT, and 1% Triton X-100 and incubated on ice for 10 min before the samples were collected.

### Caspase-3 activity.

Assay was carried out as previously described (66), with slight differences. Caspase-3 assay buffer contained 20 mM HEPES, 5 mM DTT, 2 mM EDTA, and 0.1% Triton X-100 at pH 7.4. Cell lysate (25 µg) were resuspended in the assay buffer supplemented with 25 µM Ac-DEVD-AMC (caspase-3 substrate). This mix was incubated at room temperature for 120 min. Samples were analyzed in a fluorometer equipped with a 350-nm excitation filter and 450-nm emission filter. The resulting fluorescence of 7-amino-4-methylcoumarin (AMC) from infected cells was compared with that of an untreated control, which allowed determination of the fold increase in caspase-3 activity.

### Caspase-9 activity.

Caspase-9 assay buffer contained 100 mM MOPS (morpholinepropanesulfonic acid), 10 mM DTT, 0.5 mM Na_2_EDTA, 0.1% Triton X-100 at pH 6.5, and 10% glycerol. Cell lysates (25 µg) were resuspended in the assay buffer supplemented with 10 µM Ac-LEDH-AFC (caspase-9 substrate) and incubated at 32°C for 24 h. Samples were analyzed in a fluorometer equipped with a 400-nm excitation filter and 505-nm emission filter. Fluorescence signal was treated similarly as for the caspase-3 assay.

### Caspase-8 activity.

Caspase-8 assay buffer contained 20 mM HEPES, 5 mM DTT, 2 mM Na_2_EDTA, 5% sucrose, and 0.1% Triton X-100 at pH 7.4. Cell lysates (25 µg) were resuspended in the assay buffer supplemented with 10 µM Ac-IETD-AMC (caspase-8 substrate). This mixture was incubated at 32°C for 24 h. Samples were analyzed in a fluorometer equipped with a 400-nm excitation filter and 505-nm emission filter. The fluorescence signal was treated similarly as for caspase-3 assay.

### Annexin V and PI staining.

Annexin V binding and propidium iodide (PI) uptake analyses were carried out with the Alexa Fluor 488-annexin V dead cell apoptosis kit (Invitrogen, Carlsbad, CA) according to the manufacturer’s instructions. Briefly, cells were incubated with 50 µM z-VAD-fmk, 50 µM MDL28170, 10 µM BAPTA-AM, or only dimethyl sulfoxide (DMSO) in DMEM for 1 h at 37°C where indicated. Then HEp-2 or HT-29 cells were infected with the wild-type EPEC strain or its derivatives for the indicated times. Cells were harvest with Accutase (Innovative Cell Technologies), centrifuged at 250 × *g* for 5 min, and resuspended in 1× binding buffer at a concentration of 1 × 10^7^ cells/ml. Then 100 µl of the cell suspension (1 × 10^6^ cells) was transferred to a 2-ml Eppendorf tube, and 5 µl of annexin V-Alexa Fluor 488 and 1 µl of PI (100 µg/ml) were added. The cells were gently mixed and then incubated for 15 min at room temperature in the dark. After the incubation period, 400 µl of 1× binding buffer was added to each tube, and samples were analyzed by using a FACSCalibur fluorescence-activated cell sorter (FACS) (Becton Dickinson, San Jose, CA). For each sample, 20,000 events were acquired. Annexin V-negative and PI-negative cells represented live cells. Annexin V-positive and PI-negative cells represented the early apoptotic populations. Annexin V-positive and PI-positive cells represented the late apoptotic populations. Annexin V-negative and PI-positive cells represented the necrotic populations.

### Flow cytometry analysis of cell cycle (sub-G_1_ fraction).

HEp-2 cells (1 × 10^6^ cells) infected by either the EPEC wild-type strain or its derivatives were collected, washed with cold PBS, and fixed in cold 70% ethanol (−20°C) overnight. Cells were treated with 30 µl of RNase A (1 mg/ml) and 16 µl of PI (1 mg/ml) in 956 µl of PBS. Cells were incubated for 1 h at 37°C protected from direct light. The distribution of the cell cycle phase with different DNA contents was determined with a FACSCalibur (Becton Dickinson, San Jose, CA). In each sample, 20,000 gated events were acquired. Analysis of cell cycle distribution (including sub-G_1_) was performed with the FlowJo 10.0 software.

### Measurement of ΔΨ_m_.

HEp-2 or HT-29 cells were preincubated with 2 µg/ml of mitochondrial inner membrane potential (ΔΨ_m_)-sensitive dye rhodamine 123 in DMEM (without supplements) for 30 min. Cells were washed three times with PBS and then infected with bacterial strains at an MOI of 10. After the indicated times of infection, both detached (supernatant) and adherent (trypsinized) cells were collected, and the nonadherent bacteria were removed by centrifugation (200 × *g* for 10 min at 4°C). The pellets were washed three times with PBS and then resuspended in 500 µl of PBS. The intensity of ΔΨ_m_ for at least 20,000 cells was analyzed by using a FACSCalibur.

### Subcellular fractionation of cultured cells.

The cytosolic and mitochondrial fractions of HEp-2 cells were obtained by a digitonin-based subcellular fractionation technique as described previously (67). Briefly, cells (5 × 10^6^) infected with EPEC strains at various time points were resuspended in plasma membrane permeabilization buffer (200 µg/ml digitonin, 80 mM KCl in PBS) and incubated in ice for 5 min. Lysates were centrifuged at 800 × *g* for 5 min at 4°C, and supernatant (cytosolic fraction) was collected again. The pellet was resuspended in total cell lysis buffer (50 mM Tris-HCl [pH 7.4], 150 mM NaCl, 2 mM EGTA, 2 mM Na_2_EDTA, 0.2% Triton X-100, 0.2% IGEPAL CA-630, Complete protease inhibitor) and rocked gently at 4°C for 10 min. Total lysates were centrifuged at 10,000 × *g* for 10 min at 4°C, and supernatants (mitochondria/nuclear fraction) were collected again. For the detection of released cytochrome *c* and translocated Bax, equal amounts of cytosolic and mitochondrial proteins were separated by SDS-PAGE and analyzed by immunoblotting with anti-cytochrome *c* and anti-Bax as primary antibodies and a horseradish peroxidase (HRP)-conjugated anti-isotype secondary antibody. Blots were also probed for COX IV as a control marker for the mitochondrial fractions and GAPDH as a control marker for the cytosolic fractions.

### Measurement of cytosolic Ca^2+^ levels.

Intracellular Ca^2+^ levels were measured using Fluo-4 AM staining according to the manufacturer’s protocol. Briefly, HEp-2 cells were grown in 12-well microplates and incubated with 4 µM of Fluo-4 AM in Ringer buffer (10 mM HEPES, 145 mM NaCl, 5 mM KCl, 1 mM MgCl_2_, 2 mM CaCl_2_ [pH 7.3]) in the dark for 45 min at room temperature. Cells were washed three times and incubated for a further 30 min in DMEM at 37°C to allow complete deesterification of intracellular acetocymethyl (AM) esters and then infected with bacterial strains for the indicated times. Cells were harvested with Accutase, and the mean fluorescence intensity of Fluo-4 AM was measured using a BD LSR Fortessa cytometer.

### Analyses of calpain activity.

To measure calpain activity in HEp-2 cells, the cells were preincubated with 20 µM *t*-BOC-Leu-Met-CMAC in DMEM in the dark for 45 min at 37°C. The nonfluorescent *t*-BOC-Leu-Met-CMAC enters cells and becomes an intracellular substrate for conjugation to a thiol. Subsequent intracellular proteolytic cleavage of the *t*-BOC-Leu-Met-CMAC thiol liberates and unquenches the highly fluorescent, membrane-impermeant CMAC-thiol conjugate. Loaded cells were infected with bacterial strains for the indicated lengths of time. Cells were harvested, and the cellular fluorescence intensity of the CMAC-thiol conjugate was measured using a BD LSR Fortessa cytometer.

### Immunoprecipitation and degradation assays.

HEp-2 cells grown in 100-mm petri dishes were delicately washed three times with ice-cold PBS (pH 7.4) and scraped into lysis buffer (50 mM Tris-HCl [pH 7.5], 150 mM NaCl, 1% IGEPAL CA-630, 0.5% sodium deoxycholate). Cells were collected in Eppendorf tubes at 4°C. Cells were lysed by sonication for 5 s in ice water using a Soniprep sonicator at 30% amplitude. The supernatant was centrifuged at 21,255 × *g* for 15 min at 4°C.

Five micrograms of polyclonal anti-caspase-3 (H-227) (Santa Cruz Biotechnology) antibody was incubated with cell lysates overnight at 4°C. Then protein A-agarose was added to antigen-antibody complex for 2 h at 4°C (Roche Diagnostics, Mannheim, Germany). The beads were washed three times with lysis buffer, and the immunocomplexes were resuspended in reaction buffer (250 mM Tris-HCl [pH 7.5], 1 mM DTT).

Two micrograms of immunoprecipitated procaspase-3 was mixed with an equal volume of 2× digestion buffer (0.3 mM CaCl_2_, 10 mM DTT) containing 10 µg/ml of EspC or EspC_S256I_. Reactions were carried out at 37°C for different lengths of time (15, 30, and 60 min) and then stopped by the addition of 4× Laemmli SDS-sample buffer. Samples were boiled, and proteins were separated by SDS-PAGE and analyzed by immunoblotting using anti-caspase-3 antibody (H-227; 1:1,000) or anti-EspC (1:1,000). Membranes were developed by using HRP-conjugated goat anti-rabbit IgG secondary antibody (Zymed) as indicated by the manufacturer. HRP was detected with enhanced chemiluminescence (ECL) reagent from Amersham.

### Statistical analysis.

Results are expressed as means ± standard errors of the means (SEM) from at least three independent experiments. Statistical significance was determined by *t* test and/or analysis of variance (ANOVA). A *P* value of <0.05 was considered significant.

## SUPPLEMENTAL MATERIAL

Figure S1  Confirmation of the more relevant cell death phenotypes induced by EspC on an intestinal epithelial cell line. HT-29 cells were infected with the EPEC WT, Δ*espC*, Δ*espC*/p*espC*, Δ*espC*/p*espC_S256I_*, or Δ*escN* strain at an MOI of 10 for 4 h. Mock-infected cells were used as negative controls. (A) Induction of cell death by EspC. After infection, FACS analysis via PI exclusion assay was used to observe cell death. (B) Induction of apoptosis and necrosis by EspC. FACS analysis via annexin V and PI staining was used to observe the induction of apoptosis and necrosis by flow cytometry. (C) Cleavage of procaspase-3 induced by EspC. HT-29 cells were infected with the strains as indicated at an MOI of 10 for 4 h. Infected cells were lysed, and proteins were analyzed by immunoblotting using anti-caspase-3 and anti-β-actin as primary antibodies and HRP-conjugated anti-isotype secondary antibody. The blots shown are representative of at least 3 independent experiments, and data are expressed as the mean ± SEM. (D and E) EspC is involved in the loss of mitochondrial membrane potential (ΔΨ_m_). HT-29 cells were prestained with rhodamine 123 and infected with the EPEC WT, Δ*espC*, Δ*espC*/p*espC*, Δ*espC*/p*espC_S256I_*, or Δ*escN* strain at an MOI of 10 for 4 h. Cells were analyzed for the loss of mitochondrial membrane potential (ΔΨ_m_) by flow cytometry. Statistical analysis was performed using one-way ANOVA followed by Dunnett’s multiple comparison test for comparison to the WT (*, *P* < 0.05; **, *P* < 0.01; ***, *P* < 0.001). Download Figure S1, TIF file, 1.8 MB
