# Supplementary figures and images for: EspC, an Autotransporter Protein Secreted by Enteropathogenic Escherichia coli, Causes Apoptosis and Necrosis through Caspase and Calpain Activation, Including Direct Procaspase-3 Cleavage
Source: mBio. 2016 Jun 21;7(3):e00479-16. doi: 10.1128/mBio.00479-16 (PMC4916375; doi:10.1128/mBio.00479-16)

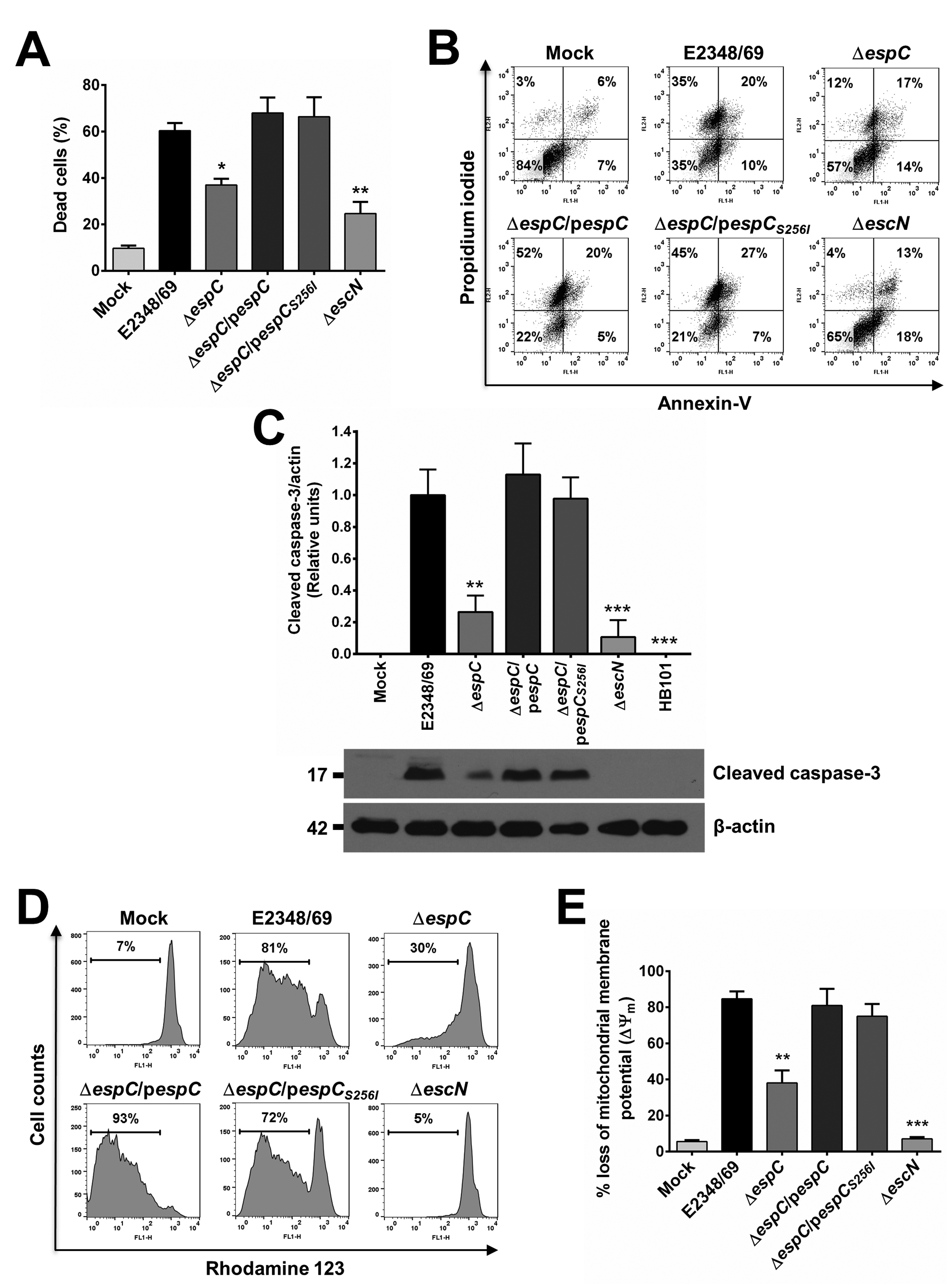

Supplement: Figure S1 — Confirmation of the more relevant cell death phenotypes induced by EspC on an intestinal epithelial cell line. HT-29 cells were infected with the EPEC WT, ΔespC, ΔespC/pespC, ΔespC/pespCS256I, or ΔescN strain at an MOI of 10 for 4 h. Mock-infected cells were used as negative controls. (A) Induction of cell death by EspC. After infection, FACS analysis via PI exclusion assay was used to observe cell death. (B) Induction of apoptosis and necrosis by EspC. FACS analysis via annexin V and PI staining was used to observe the induction of apoptosis and necrosis by flow cytometry. (C) Cleavage of procaspase-3 induced by EspC. HT-29 cells were infected with the strains as indicated at an MOI of 10 for 4 h. Infected cells were lysed, and proteins were analyzed by immunoblotting using anti-caspase-3 and anti-β-actin as primary antibodies and HRP-conjugated anti-isotype secondary antibody. The blots shown are representative of at least 3 independent experiments, and data are expressed as the mean ± SEM. (D and E) EspC is involved in the loss of mitochondrial membrane potential (ΔΨm). HT-29 cells were prestained with rhodamine 123 and infected with the EPEC WT, ΔespC, ΔespC/pespC, ΔespC/pespCS256I, or ΔescN strain at an MOI of 10 for 4 h. Cells were analyzed for the loss of mitochondrial membrane potential (ΔΨm) by flow cytometry. Statistical analysis was performed using one-way ANOVA followed by Dunnett’s multiple comparison test for comparison to the WT (*, P < 0.05; **, P < 0.01; ***, P < 0.001). Download [file mbo003162852sf1.tif]
